# Preparation of Chitosan-Composite-Film-Supported Copper Nanoparticles and Their Application in 1,6-Hydroboration Reactions of *p*-Quinone Methides

**DOI:** 10.3390/molecules27227962

**Published:** 2022-11-17

**Authors:** Shuhan Chen, Wei Wen, Xue Zhao, Zelang Zhang, Weishuang Li, Yaoyao Zhang, Bojie Li, Lei Zhu

**Affiliations:** 1 School of Chemistry and Materials Science, Hubei Engineering University, Xiaogan 432000, China; 2 School of Materials Science and Engineering, Hubei University, Wuhan 430062, China

**Keywords:** copper nanoparticles, chitosan composite film, hydroboration reactions, *p*-quinone methides, organoboron compounds

## Abstract

Here, we describe the preparation of copper nanoparticles that are stabilized on a chitosan composite film (CP@Cu). This material could catalyze the 1,6-hydroboration reactions of *p*-quinone methides with B_2_pin_2_ as a boron source under mild conditions. This reaction exhibited very good functional group compatibility, and the organoboron compounds that were formed could easily be converted into corresponding hydroxyl products with good to excellent yields. This newly developed methodology provides an efficient and sequential pathway for the synthesis of *gem*-disubstituted methanols.

## 1. Introduction

Not only do organoboron compounds exist in a wide range of active molecules, natural products, and materials [1,2,3], but they are also the key intermediates in the synthesis of many functional chemicals [4,5]. For these reasons, in recent years, a series of methodologies—especially transition metal catalysis—have been developed for the synthesis of organoboron compounds [6,7,8], which have become more and more important [9,10,11,12]. Copper catalysts are increasingly favored by organic chemists due to their low cost, lower toxicity, and solid performance [13,14]. In previous work, copper-catalyzed hydroboration reactions of unsaturated compounds were widely studied, and this is also a common method for constructing C-B bonds [15,16,17,18]. However, examples reported here involved the use of strong bases and specifically designed ligands, which reduced the reaction economy. Thus, it is necessary to explore alternative highly active and sustainable copper-catalyzed hydroboration reactions of unsaturated compounds.

**Scheme 1:**
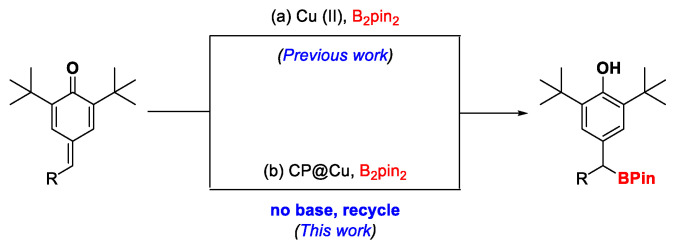
Cu(II)-catalyzed 1,6-hydroboration of *p*-quinone methides.

Metallic nanoparticles have been widely used in various reactions with the continuous development of organic synthetic chemistry over recent decades [19,20,21,22]. However, to the best of our knowledge, though they are some of the most widely used metallic nanoparticles, copper nanoparticles are rarely used to catalyze the hydroboration of unsaturated compounds [23,24]. In particular, with *p*-quinone methides, as a class of intermediates with a wide range of applications in organic synthesis [25,26,27,28], the 1,6-hydroboration products can be well transformed into *gem*-disubstituted methanols under certain conditions; they are widely spread throughout nature and are the core skeletons of many biologically active molecules and natural products [29,30,31,32,33]. So far, there have only been a few reports in the literature on the copper-catalyzed 1,6-boron addition reaction of *p*-quinone methides, and these reports were mainly focused on Cu(I)-catalyzed reactions [34,35]. In our previous work, we used Cu(OH)_2_ as a catalyst to investigative the 1,6-hydroboration reaction of *p*-quinone methides, and good functional group compatibilities and reaction yields were obtained (Scheme 1a) [36]. Based on the results of our previous research, we found that when chitosan-supported copper nanoparticles were used as a heterogeneous catalyst, construct C-B bonds [37] and C-Si bonds could be constructed with a high efficiency [38]. Therefore, in this work, we hope to use chitosan-supported copper nanoparticles as a heterogeneous catalyst and *p*-quinone methides as substrates to study the 1,6-hydroboration reactions. In comparison with previous work, the biggest advantage of this work is the avoidance of the participation of bases in the reactions and the recycling of the catalyst to increase its utilization rate (Scheme 1b).

## 2. Results and Discussion

The initial experiments commenced with *p*-quinone methide **1a** as a model substrate. CP@Cu (6 mol%) was used as a catalyst by using B_2_Pin_2_ (1.2 equiv.) as the boron source in the reactions. Firstly, the various organic solvents were investigated, and considering the role of protons, the whole reaction was started with ethanol (2.5 mL) as the solvent. However, no reaction happened (Table 1, Entry 1). When DCM and THF were used as solvents and MeOH (2 equiv.) was used as an additive, reactions were still not observed (Table 1, Entries 2–3). To our delight, when MeCN was used as the solvent and MeOH (2 equiv.) was used as an additive, the reaction was able to take place, and the desired product **2a** was smoothly obtained. It was confirmed that further oxidation of **2a** gave the corresponding *gem*-disubstituted methanol product **3a** by using NaBO_3_·4H_2_O as an oxidant. (Table 1, Entry 4). Continuing to use acetone as the solvent and MeOH (2 equiv.) as an additive further promoted the occurrence of the reaction, and the target product **3a** could be obtained with 70% yield (Table 1, Entry 5). Since water was a green solvent in this reaction, we added water (2 equiv.) to the reaction as an additive; unfortunately, the reaction did not happen (Table 1, Entry 6). As far as we know, in organic synthesis reactions, the use of mixed solvents could sometimes greatly improve the efficiency of the whole reaction. Therefore, in order to further improve the yield, we considered using acetone and H_2_O as mixed solvents to carry out the reaction, and the ratios of the solvents were screened (Table 1, Entries 7–10). When the ratio of acetone to H_2_O was 4:1, the reaction had the highest rate of conversion, and it occurred almost completely; the final target product could be obtained with 98% yield (Table 1, Entry 7). When the weight of H_2_O in the mixed solvents was continually increased, it was found that the conversion rate of the reaction decreased as the proportion of water increased; even when the ratio of acetone to H_2_O was reversed to 1:4, the reaction hardly occurred, and only trace amounts of product could be detected (Table 1, Entry 10). In order to verify the importance of the CP@Cu in the reactions, we performed a control experiment, and no reaction occurred without any CP@Cu, which proved that the catalyst was indispensable in these reactions (Table 1, Entry 11). Finally, the reaction time was also investigated. Even if the reaction time was shortened to 1 h, the reaction still occurred efficiently and produced the target product with 93% yield (Table 1, Entry 12). Thus, through a series of optimizations of the conditions, the optimal conditions in this research were found to be 6 mol% of CP@Cu as a catalyst and 1.2 equiv. of B_2_Pin_2_ as a boron source, and the whole reaction was conducted in 2.5 mL of mixed solvents (acetone:H_2_O = 4:1) at room temperature for 2 h (Table 1, Entry 7).

**Figure 1:**
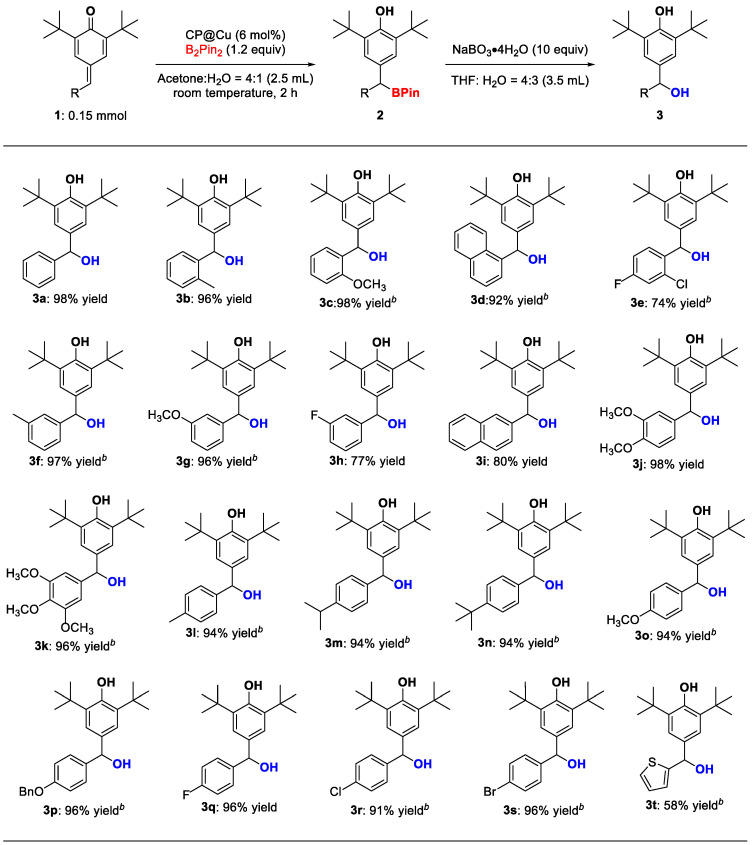
Screening of the scope of the substrates. Reaction conditions: *^a^* **1a** (0.15 mmol), B_2_Pin_2_ (0.18 mmol), CP@Cu (5.0 mg, 0.009 mmol), acetone:H_2_O = 4:1 (2.5 mL) in room temperature for 2 h. *^b^* 12 h.

With the optimal conditions in hand, we continued to examine the universality of the reaction, and the results are summarized in Figure 1. Firstly, the effects of substituents at the *ortho*-position of the benzene ring on the reaction were investigated. For electron-donating substituents, such as methyl and methoxy, the desired target product could be obtained with excellent reaction yields (**3b**–**3c**, 96–98% yields). The whole reaction could still proceed smoothly and achieved the corresponding products with satisfactory yields when the more conjugated 2-substituted naphthyl was selected as the substituent instead of phenyl (**3d**, 92% yield). Although the electron-withdrawing substituents at the *ortho*-position had a certain effect on the reaction, the desired product was still obtained with a good yield (**3e**, 74% yield).

Next, we investigated the reactivity of the substituents at the *meta*-position of the benzene ring. From the reaction results summarized in Figure 1, the electron-donating substituents had a good effect on the reaction, and the desired products could be obtained with an almost equivalent yield (**3f**–**3g**, 96–97% yields). However, when an electron-withdrawing substituent was used, such as fluorine, the reaction yield was reduced to some extent (**3h**, 77% yield). To our delight, when the benzene ring had multiple substituents, such as naphthyl, dimethoxy, or even trimethoxy, the reaction could still occur well, and good to excellent reaction yields could be obtained (**3i**–**3k**, 80–96% yields). We also investigated the reactivity of *para*-substituents on the benzene ring; both electron-donating substituents (methyl, isopropyl, *tert*-butyl, methoxy, and benzyloxy) and electron-withdrawing substituents (fluorine, chlorine, bromine) had little effect on the reaction (**3l**–**3s**, 91–96% yields). Finally, we investigated thiophene, and although the target product could be obtained only with a moderate yield, the reaction still proved that the catalyst had good functional group compatibility (**3t**, 58% yield).

**Figure 2:**
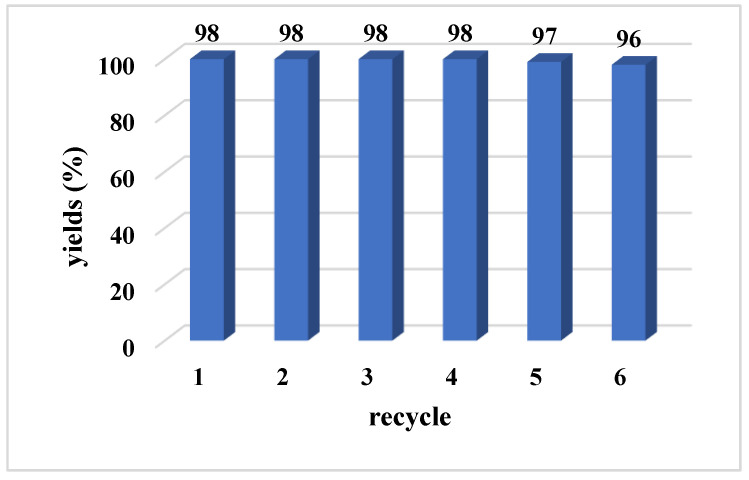
The recycling experiments.

Considering that this could be a heterogeneous catalyst in this reaction, it is necessary to identify the reusability and stability of catalyst. It was demonstrated that when the reaction was completed, the CP@Cu catalyst could be easily recycled with a simple operation. The catalytic activity stayed almost the same after experimenting with recycling the catalyst six times, and the yield was still up to 96% even in the sixth experiment, so the catalyst has the advantage of being recyclable (Figure 2).

**Figure 3:**
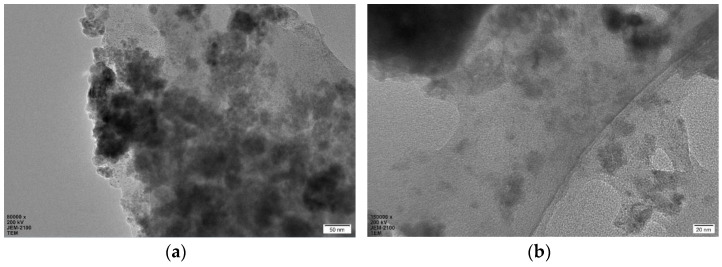
TEM images of (**a**) CP@Cu at 60 nm and (**b**) CP@Cu at 20 nm.

The Cu nanoparticles supported on a chitosan–PVA composite are shown in Figure 3. As observed, the dark spherical particles in the red circles are the CuNPs, which are uniformly dispersed in the CP matrix. The particle sizes ranged from 2 to 4 nm, showing that the Cu nanoparticles were uniformly distributed on the chitosan–PVA composite. In addition, no aggregation of CuNPs was noticed, which confirmed that the CP matrix is a good stabilizing agent for the synthesis of CuNPs. The good dispersion of CuNPs into the CP matrix enhanced their performance during the catalytic process [39].

**Figure 4:**
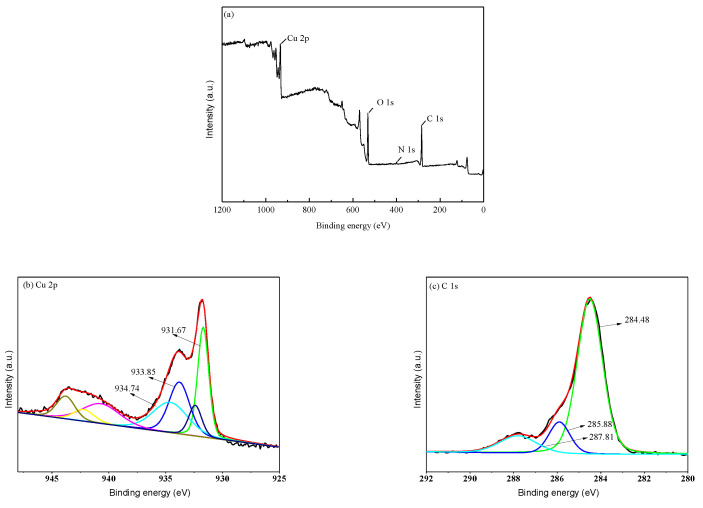
High-resolution XPS spectra of (**a**) CP@Cu after metal adsorption; (**b**) high-resolution Cu 2p spectra; (**c**) high-resolution C 1s spectra after adsorption.

The full-scan XPS spectrum showed that the major elements of the Cu nanoparticles supported on the chitosan–PVA composite were O, C, and N (Figure 4a). This is consistent with the chemical structures of chitosan and PVA, which are rich in the functional groups of –NH–C=O, –NH_2_, and –OH. The presence of a Cu 2p peak in the Cu-loaded PVA–CS nanofiber membrane proved the adsorption of Cu(II) onto the adsorbent. The spectrum of the adsorbent after copper adsorption showed peaks at 932.67, 933.85, and 934.74 eV, which corresponded to Cu 2p (Figure 4b). The C 1s spectra of the Cu nanoparticles supported on the chitosan–PVA composite showed peaks at 284.48, 285.88, and 287.81 eV after the adsorption of Cu(II), indicating the involvement of the functional groups in the adsorption of Cu(II) onto the adsorbent (Figure 4c) [40,41].

## 3. Materials and Methods

### 3.1. Materials

Chitosan (degree of deacetylation ≥95%, viscosity 100–200 MPa·s) was purchased directly from Aladdin (Shanghai, China), poly (vinyl alcohol) (M_w_ 120 kDa) was purchased from Sinopharm Chemical Reagent Co., Ltd. (Beijing, China), B_2_pin_2_ was purchased from Energy Chemical (Shanghai, China), and CuCl_2_·2H_2_O was purchased from Sinopharm Chemical Reagent Co., Ltd. (Beijing, China). All *p*-quinone methides were obtained smoothly through reactions between aldehydes and variously substituted 2,6-di-*tert*-butylphenol. Chitosan/poly (vinyl alcohol)-composite-film-supported copper nanoparticles (CP@Cu NPs) were prepared according to the method reported in the literature [39].

### 3.2. Analytical Methods

Nuclear magnetic resonance (NMR) spectra were recorded on a Bruker Avance III 400 MHz spectrometer (Karlsruhe, Germany) operating at 400 for ^1^H and 100 MHz for ^13^C NMR in CDCl_3_ unless otherwise noted. CDCl_3_ served as the internal standard (δ = 7.26 ppm) for ^1^H NMR and (δ = 77.0 ppm) for ^13^C NMR. The data for ^1^H NMR are reported as follows (See the Supplementary Materials): chemical shift (ppm, scale), multiplicity (s = singlet, d = doublet, t = triplet, q = quartet, m = multiplet and/or multiplet resonances, br = broad), coupling constant (Hz), and integration. The data for ^13^C NMR are reported in terms of the chemical shift (ppm, scale), multiplicity, and coupling constant (Hz). The purification of products was accomplished by using flash column chromatography on silica gel (200–300 mesh) or preparative TLC. The weight percentage and metal leaching of copper were determined by inductively coupled plasma–optical emission spectroscopy (ICP-OES) (PerkinElmer, Waltham, MA, USA) analysis.

An X-ray photoelectron spectroscopy (XPS) analysis was performed with a Thermo Fisher ESCALAB250Xi spectrometer (Thermo Fisher Scientific, Waltham, MA, USA) by using monochromatized Al-Ka radiation at a detection angle of 30°. The photon energy was 1486.6 eV. A pass energy of 30 eV was used for high-resolution scans in a valence band analysis. The test area size was 500 um. The binding energy of all spectra was determined by using binding energy correction with respect to polluting carbon (C 1s, 284.6 or 284.8eV). The spectra were collected over a range of 0–1486.6 eV, and the high-resolution spectra of C 1s and Cu 2p regions were provided. The Shirley background and Gaussian/Lorentzian functions were used to fit the peaks.

Transmission electron microscopy (TEM) was used to observe the morphology on a Jeol 2100f instrument (JEOL, Tokyo, Japan). Samples were prepared for TEM analysis by placing a drop of a sample of a particle suspension on a copper grid and quickly wicking away the solution with filter paper.

### 3.3. General Procedure for the Preparation of CP@Cu NPs

According to a report in the literature [39], 200 mg of chitosan powder was dissolved in 10 mL of acetic acid solution (2%, *v*/*v*) and stirred at room temperature for 5 h. At the same time, 400 mg of poly (vinyl alcohol) was dissolved in 10 mL of water and stirred at 80 °C for 12 h. The two solutions obtained were mixed and stirred at room temperature for another 0.5 h; then, 32 μL of glutaraldehyde solution (25%, *w*/*w*) was added, and stirring was continued for 5 min. In order to form the chitosan/poly (vinyl alcohol) composite film, the mixed solution described above was transferred to a Petri dish and dried at 40 °C for 12 h. After completion of this procedure, 0.1 mol/L of NaOH solution was added to the above composite film and allowed to soak for 5 min; then, this was washed until it was neutral by using water and dried over 12 h at 40 °C. After immersing the composite film in 0.2 mol/L CuCl_2_ solution for 2.5 h, the excess Cu^2+^ and Cl^−^ were removed by washing with water, and then drying took place at 40 °C for 12 h. Finally, the chitosan/poly (vinyl alcohol)-composite-film-supported copper nanoparticles (CP@Cu NPs) were obtained by reducing with 0.05 mol/L of NaBH_4_ solution, and they were then submitted for ICP analysis. The copper loading of the CP@Cu NPs was found to be 1.78 mmol/g.

### 3.4. Recycling and Reuse of CP@Cu NPs

To demonstrate the recyclability of the CP@Cu NPs, the addition of a boron conjugate was repeated six times with the same composite film. The initial amount of catalyst was 5 mg (6 mol % Cu loading). Reactions were carried out under standard conditions. After the completion of the reaction, the catalyst was filtered off, washed with acetone, and then dried at 50 °C before the next run.

## 4. Conclusions

In conclusion, we have reported the preparation of a copper nanoparticle stabilized on chitosan composite film (CP@Cu) and its application for catalyzing the 1,6-hydroboration reaction of *p*-quinone methides with B_2_pin_2_ as a boron source. The conditions of the whole reaction were very mild, and no additional bases were needed. This newly developed methodology showed very good functional group compatibility and reactivity (20 examples, up to 98% yield). The organoboron products that were formed could be easily and directly oxidized to the corresponding hydroxyl products with good to excellent yields. In addition, the recycling experiments evidenced that this catalyst still showed good reactivity after being recycled six times (>96% yield), which proved that the catalyst had good reusability and stability.

## Figures and Tables

**Table 1:** Reaction condition optimizations *^a^*.

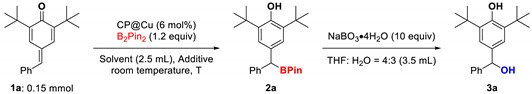					
Entries	CP@Cu	Solvent	Additive	T (h)	Yields (%) *^b^*
1	6 (mol%)	EtOH	-	2	N.R.
2	6 (mol%)	DCM	MeOH (2 equiv.)	2	N.R.
3	6 (mol%)	THF	MeOH (2 equiv.)	2	N.R.
4	6 (mol%)	MeCN	MeOH (2 equiv.)	2	34
5	6 (mol%)	Acetone	MeOH (2 equiv.)	2	70
6	6 (mol%)	Acetone	H_2_O (2 equiv.)	2	N.R.
7	6 (mol%)	Acetone:H_2_O (4:1)	-	2	98
8	6 (mol%)	Acetone:H_2_O (2:1)	-	2	84
9	6 (mol%)	Acetone:H_2_O (1:2)	-	2	59
10	6 (mol%)	Acetone:H_2_O (1:4)	-	2	<5
11	-	Acetone:H_2_O (4:1)	-	2	N.R.
12	6 (mol%)	Acetone:H_2_O (4:1)	-	1	93

Reaction conditions: *^a^* **1a** (0.15 mmol), B_2_Pin_2_ (0.18 mmol), CP@Cu (5.0 mg, 0.009 mmol), solvents (2.5 mL) at room temperature. *^b^* Isolated yield. N.R. = No reaction.

## Supplementary Materials

The following supporting information can be downloaded at: https://www.mdpi.com/article/10.3390/molecules27227962/s1, characterization data, and spectra for the ^1^H and ^13^C NMR of products **3a**–**3t**. Citation: [34,36].
