# Supplementary material for: Preparation of Chitosan-Composite-Film-Supported Copper Nanoparticles and Their Application in 1,6-Hydroboration Reactions of p-Quinone Methides
Source: Molecules. 2022 Nov 17;27(22):7962. doi: 10.3390/molecules27227962 (PMC9695593; doi:10.3390/molecules27227962)
Supplement: Supplementary file 1 [file molecules-27-07962-s001.zip › molecules-1985314-supplementary.pdf]

supporting information

Preparation of Chitosan Composite Film Supported Copper Nanoparticles and Its Application in 1,6-Hydroboration Reactions of *p*-Quinone Methides

Shuhan Chen [a,b], Wei Wen [a,b], Xue Zhao [a,b], Zelang Zhang [a,b], Weishuang Li [a,*], Yaoyao Zhang [a], Bojie Li [a,*], Lei Zhu [a,b,*]

[a] *School of Chemistry and Materials Science, Hubei Engineering University, Xiaogan 432000*

[b] *School of Materials Science and Engineering, Hubei University, Wuhan 430062, China*

* *Corresponding author. E-mail: Lei.zhu@hbeu.edu.cn (L. Z.), liweishuang706@hbeu.edu.cn (W. L.), boji.li@hbeu.edu.cn (B. L.).*

Contents

General Procedure for 1,6-Hydroboration Reactions of *p*-Quinone Methides .. S-2

References S-6

NMR Spectral of Products S-7

General Procedure for 1,6-Hydroboration Reactions of *p*-Quinone Methides

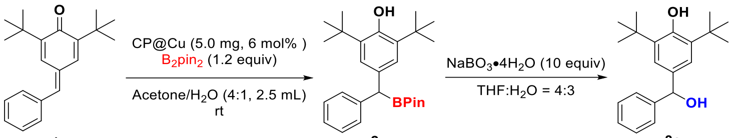

General procedure: To a 3.0 mL of vial equipped with a magnetic stirring bar were added 4-benzylidene-2,6-di-*tert*-butylcyclohexa-2,5-dien-1-one **1a** (0.15 mmol), B_2pin_2 (0.18 mmol) and CP@Cu NPs (5.0 mg, 0.009 mmol), then mixture of solvents (2.5 mL, Acetone: H_2O = 4:1) was added to the system. The whole reaction was stirred at room temperature and monitored by TLC, after completion of the reaction, filter through a celite pad, the mixture was extracted with EtOAc (15 mL × 3), washed with brine, separated and dried over anhydrous $MgSO_4$, the solvents were removed under reduced pressure to obtained the crude product **2a**. To the above crude product in the flask were added $NaBO_3$•$4H_2O$ (243.5 mg, 10 equiv) and solvents (3.5 mL, THF: H_2O = 4:3), the whole reaction continued to stir at room temperature for 4 h, when the reaction finished, extracted with EA (15 mL × 3), combined the organic phase and washed with brine (10 mL), separated and dried over anhydrous $MgSO_4$. The solvents were removed under vacuum and the desired product **3a** (98% yield) was obtained as a white solid after purification by silica gel chromatography. ^{1}H NMR (400 MHz, $CDCl_3$) δ 7.42 – 7.37 (m, 2H), 7.35 – 7.30 (m, 2H), 7.27 – 7.21 (m, 1H), 7.16 (s, 2H), 5.81 – 5.68 (m, 1H), 5.18 (s, 1H), 2.22 (d, *J* = 3.1 Hz, 1H), 1.40 (s, 18H); ^{13}C NMR (100 MHz, $CDCl_3$) δ 153.34, 144.13, 135.86, 134.55, 128.35, 127.26, 126.41, 123.67, 76.77, 34.41, 30.27. [1]

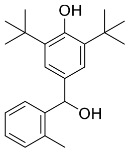

3b

2,6-di-*tert*-Butyl-4-(hydroxy(*o*-tolyl)methyl)phenol 3b:[1] ^{1}H NMR (400 MHz, $CDCl_3$) δ 7.60 – 7.54 (m, 1H), 7.27 – 7.21(m, 1H), 7.20 – 7.14 (m, 1H), 7.13 – 7.08 (m, 3H), 5.92 – 5.88 (m, 1H), 5.17 (s, 1H), 2.25 (s, 3H), 2.15 – 2.09 (m, 1H), 1.39 (s, 18H); ^{13}C NMR (100 MHz, $CDCl_3$) δ 153.29, 141.99, 135.78, 135.10, 133.47, 130.35, 127.17, 126.07, 125.73, 124.09, 73.66, 34.39, 30.28,

19.53.

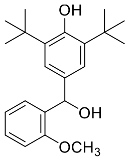

3c

2,6-di-*tert*-Butyl-4-(hydroxy(2-methoxyphenyl)methyl)phenol 3c:[1] ^{1}H NMR (400 MHz, $CDCl_3$) δ 7.27 – 7.18 (m, 4H), 6.96 – 6.91 (m, 1H), 6.90 – 6.85 (m, 1H), 5.99 (d, *J* = 3.7 Hz, 1H), 5.14 (s, 1H), 3.83 (s, 3H), 3.02 (d, *J* = 4.2 Hz, 1H), 1.41 (s, 18H); ^{13}C NMR (100 MHz, $CDCl_3$) δ 156.73, 153.03, 135.49, 133.65, 132.45, 128.42, 127.66, 123.66, 120.82, 110.61, 72.69, 55.40, 34.41, 30.35.

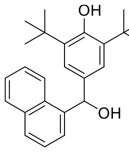

3d

2,6-di-*tert*-Butyl-4-(hydroxy(naphthalen-1-yl)methyl)phenol 3d: [2] ^{1}H NMR (400 MHz, $CDCl_3$) δ 8.14 – 8.07 (m, 1H), 7.90 – 7.84 (m, 1H), 7.83 – 7.76 (m, 1H), 7.69 – 7.63 (m, 1H), 7.53 – 7.44 (m, 3H), 7.22 (s, 2H), 6.51 (d, *J* = 3.5 Hz, 1H), 5.18 (s, 1H), 2.24 (d, *J* = 3.8 Hz, 1H), 1.38 (s, 18H); ^{13}C NMR (100 MHz, $CDCl_3$) δ 153.35, 139.31, 135.80, 133.80, 133.60, 130.81, 128.68, 128.09, 125.93, 125.44, 125.36, 124.07, 124.04, 124.00, 73.73, 34.36, 30.23.

2,6-di-*tert*-Butyl-4-((2-chloro-4-fluorophenyl)(hydroxy)methyl)phenol 3e: ^{1}H

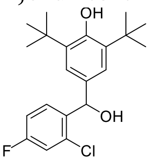

3e

NMR (400 MHz, $CDCl_3$) δ 7.66 (dd, *J* = 8.7, 6.2 Hz, 1H), 7.19 (s, 2H), 7.16 – 6.97 (m, 2H), 6.10 (d, *J* = 2.8 Hz, 1H), 5.22 (s, 1H), 2.33 (d, *J* = 3.0 Hz, 1H), 1.42 (s, 18H); ^{13}C NMR (100 MHz, $CDCl_3$) δ 161.55 (d, *J* = 247.3 Hz), 153.48, 137.60 (d, *J* = 3.4 Hz), 135.89, 132.90 (d, *J* = 10.2 Hz), 132.77, 128.80 (d, *J* = 8.7 Hz), 123.69, 116.69 (d, *J* = 24.6 Hz), 114.19 (d, *J* = 20.8 Hz), 72.63, 34.40, 30.25.

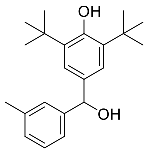

3f

2,6-di-*tert*-Butyl-4-(hydroxy(*m*-tolyl)methyl)phenol 3f:[1] ^{1}H NMR (400 MHz, $CDCl_3$) δ 7.26 – 7.16 (m, 5H), 7.10 – 7.05 (m, 1H), 5.74 (d, *J* = 2.6 Hz, 1H), 5.19 (s, 1H), 2.36 (s, 3H), 2.15 (d, *J* = 2.9 Hz, 1H), 1.42 (s, 18H); ^{13}C NMR (100 MHz, $CDCl_3$) δ 153.31, 144.06, 137.96, 135.82, 134.59, 128.25, 128.03, 127.08, 123.63, 123.42, 76.82, 34.40, 30.27, 21.54.

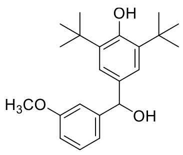

3g

2,6-di-*tert*-Butyl-4-(hydroxy(3-methoxyphenyl)methyl)phenol 3g:[1] ^{1}H NMR (400 MHz, $CDCl_3$) δ 7.29 – 7.22 (m, 1H), 7.17 (s, 2H), 7.03 – 6.92 (m, 2H), 6.86 – 6.74 (m, 1H), 5.75 – 5.71 (m, 1H), 5.18 (s, 1H), 3.80 (s, 3H), 2.19 (d, *J* = 2.9 Hz, 1H), 1.41 (s, 18H); ^{13}C NMR (100 MHz, $CDCl_3$) δ

159.63, 153.37, 145.82, 135.86, 134.39, 129.36, 123.62, 118.82, 112.76, 111.90, 76.68, 55.25, 34.40, 30.27.

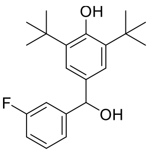

3h

2,6-di-*tert*-Butyl-4-((3-fluorophenyl)(hydroxy)methyl)phenol 3h:[1] ^{1}H NMR (400 MHz, $CDCl_3$) δ 7.31 – 7.21 (m, 1H), 7.18 – 7.05 (m, 4H), 6.96 – 6.89 (m, 1H), 5.73 (d, J = 2.6 Hz, 1H), 5.21 (s, 1H), 2.22 (d, J = 2.5 Hz, 1H), 1.40 (s, 18H); ^{13}C NMR (100 MHz, $CDCl_3$) δ 162.91 (d, J = 244.0 Hz), 153.57, 146.78 (d, J = 6.8 Hz), 136.06, 134.11, 129.78 (d, J = 8.1 Hz), 123.67, 121.95 (d, J = 2.8 Hz), 114.01 (d, J = 21.2 Hz), 113.28 (d, J = 21.9 Hz), 76.18 (d, J = 1.9 Hz), 34.42, 30.25.

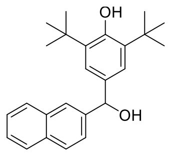

3i

2,6-di-*tert*-Butyl-4-(hydroxy(naphthalen-2-yl)methyl)phenol 3i:[1] ^{1}H NMR (400 MHz, $CDCl_3$) δ 7.96 – 7.89 (m, 1H), 7.89 – 7.74 (m, 3H), 7.54 – 7.39 (m, 3H), 7.22 (s, 2H), 5.94 (d, J = 2.9 Hz, 1H), 5.20 (s, 1H), 2.26 (d, J = 3.1 Hz, 1H), 1.41 (s, 18H); ^{13}C NMR (100 MHz, $CDCl_3$) δ 153.43, 141.50, 135.95, 134.42, 133.31, 132.77, 128.10, 128.05, 127.65, 126.03, 125.75, 124.88, 124.69, 123.79, 76.90, 34.40, 30.26.

2,6-di-*tert*-Butyl-4-((3,4-dimethoxyphenyl)(hydroxy)methyl)phenol 3j:[1] ^{1}H NMR

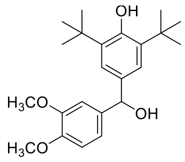

3j

(400 MHz, $CDCl_3$) δ 7.17 (s, 2H), 7.00 (d, J = 1.9 Hz, 1H), 6.93 – 6.87 (m, 1H), 6.83 (d, J = 8.3 Hz, 1H), 5.75 – 5.71 (m, 1H), 5.19 (s, 1H), 3.87 (s, 6H), 2.19 – 2.11 (m, 1H), 1.41 (s, 18H); ^{13}C NMR (100 MHz, $CDCl_3$) δ 153.31, 148.86, 148.19, 136.77, 135.83, 134.50, 123.57, 118.82, 110.84, 109.68, 76.50, 55.92, 55.85, 34.40, 30.27.

2,6-di-*tert*-Butyl-4-(hydroxy(3,4,5-trimethoxyphenyl)methyl)phenol 3k:[1] 1H

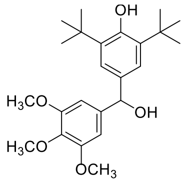

3k

NMR (400 MHz, $CDCl_3$) δ 7.19 (s, 2H), 6.65 (s, 2H), 5.70 (d, J = 2.7 Hz, 1H), 5.21 (s, 1H), 3.84 (s, 6H), 3.83 (s, 3H), 2.21 (d, J = 3.0 Hz, 1H), 1.42 (s, 18H); ^{13}C NMR (100 MHz, $CDCl_3$) δ 153.44, 153.13, 139.73, 137.04, 135.90, 134.18, 123.63, 103.48, 76.80, 60.87, 56.10, 34.42, 30.27.

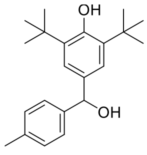

3l

2,6-di-*tert*-Butyl-4-(hydroxy(*p*-tolyl)methyl)phenol 3l:[1] ^{1}H NMR (400 MHz, $CDCl_3$) δ 7.30 (d, *J* = 8.1 Hz, 2H), 7.19 (s, 2H), 7.16 (d, *J* = 7.6 Hz, 2H), 5.78 – 5.72 (m, 1H), 5.19 (s, 1H), 2.35 (s, 3H), 2.17 (d, *J* = 3.0 Hz, 1H), 1.43 (s, 18H); ^{13}C NMR (100 MHz, $CDCl_3$) δ 153.26, 141.29, 136.86, 135.82, 134.69, 129.04, 126.36, 123.53, 76.66, 34.40, 30.29, 21.16.

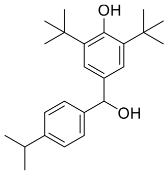

3m

2,6-di-*tert*-Butyl-4-(hydroxy(4-isopropylphenyl)methyl)phenol 3m:[1] ^{1}H NMR (400 MHz, $CDCl_3$) δ 7.34 (d, *J* = 8.1 Hz, 2H), 7.22 (d, *J* = 8.3 Hz, 2H), 7.21 (s, 2H), 5.79 – 5.73 (m, 1H), 5.20 (s, 1H), 3.05 – 2.83 (m, 1H), 2.20 (d, *J* = 4.0 Hz, 1H), 1.44 (s, 18H), 1.26 (d, *J* = 6.9 Hz, 6H); ^{13}C NMR (100 MHz, $CDCl_3$) δ 153.26, 147.93, 141.67, 135.81, 134.63, 126.44, 123.54, 76.71, 34.42, 33.84, 30.30, 24.06.

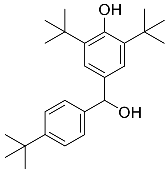

3n

2,6-di-*tert*-Butyl-4-((4-(*tert*-butyl)phenyl)(hydroxy)methyl)phenol 3n:[1] ^{1}H NMR (400 MHz, $CDCl_3$) δ 7.42 – 7.29 (m, 4H), 7.21 (s, 2H), 5.78 – 5.74 (m, 1H), 5.19 (s, 1H), 2.19 (d, *J* = 2.9 Hz, 1H), 1.43 (s, 18H), 1.33 (s, 9H); ^{13}C NMR (100 MHz, $CDCl_3$) δ 153.26, 150.17, 141.27, 135.80, 134.58, 126.15, 125.30, 123.51, 76.66, 34.52, 34.41, 31.40, 30.30.

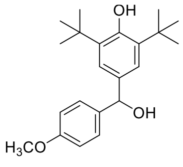

3o

2,6-di-*tert*-Butyl-4-(hydroxy(4-methoxyphenyl)methyl)phenol 3o:[1] ^{1}H NMR (400 MHz, $CDCl_3$) δ 7.31 (d, *J* = 8.4 Hz, 2H), 7.16 (s, 2H), 6.87 (d, *J* = 8.6 Hz, 2H), 5.73 (d, *J* = 2.0 Hz, 1H), 5.17 (s, 1H), 3.80 (s, 3H), 2.12 (d, *J* = 3.1 Hz, 1H), 1.41 (s, 18H); ^{13}C NMR (100 MHz, $CDCl_3$) δ 158.79, 153.23, 136.46, 135.81, 134.71, 127.71, 123.50, 113.71, 76.34, 55.29, 34.40, 30.28.

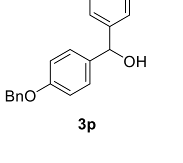

3p

4-((4-(Benzyloxy)phenyl)(hydroxy)methyl)-2,6-di-*tert*-butylphenol 3p:[1] ^{1}H NMR (400 MHz, $CDCl_3$) δ 7.49 – 7.28 (m, 7H), 7.18 (s, 2H), 7.01 – 6.94 (m, 2H), 5.75 (d, *J* = 2.4 Hz, 1H), 5.19 (s, 1H), 5.08 (s, 2H), 2.15 (d, *J* = 3.2 Hz, 1H), 1.43 (s, 18H); ^{13}C NMR (100 MHz, $CDCl_3$) δ 158.03, 153.26, 137.05, 136.75, 135.82, 134.69, 128.59, 127.96, 127.75, 127.49, 123.53, 114.71, 76.34, 70.05, 34.41, 30.29.

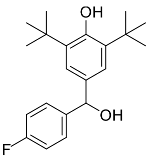

3q

2,6-di-*tert*-Butyl-4-((4-fluorophenyl)(hydroxy)methyl)phenol 3q:[1] ^{1}H NMR (400 MHz, CDCl$_3$) δ 7.41 – 7.33 (m, 2H), 7.15 (s, 2H), 7.06 – 6.99 (m, 2H), 5.76 (d, *J* = 2.6 Hz, 1H), 5.22 (s, 1H), 2.22 (d, *J* = 2.9 Hz, 1H), 1.42 (s, 18H); ^{13}C NMR (100 MHz, CDCl$_3$) δ 162.01 (d, *J* = 243.6 Hz), 153.45, 139.86 (d, *J* = 3.0 Hz), 135.99, 134.42, 128.06 (d, *J* = 8.0 Hz), 123.60, 115.10 (d, *J* = 21.4 Hz), 76.11, 34.41, 30.26.

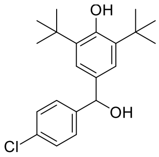

3r

2,6-di-*tert*-Butyl-4-((4-chlorophenyl)(hydroxy)methyl)phenol 3r:[1] ^{1}H NMR (400 MHz, CDCl$_3$) δ 7.40 – 7.28 (m, 4H), 7.13 (s, 2H), 5.74 (d, *J* = 2.4 Hz, 1H), 5.22 (s, 1H), 2.19 (d, *J* = 3.0 Hz, 1H), 1.42 (s, 18H); ^{13}C NMR (100 MHz, CDCl$_3$) δ 153.53, 142.59, 136.06, 134.21, 132.86, 128.42, 127.76, 123.62, 76.13, 34.41, 30.25.

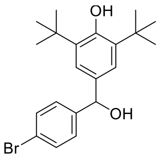

3s

4-((4-Bromophenyl)(hydroxy)methyl)-2,6-di-*tert*-butylphenol 3s:[1] ^{1}H NMR (400 MHz, CDCl$_3$) δ 7.46 (d, *J* = 8.4 Hz, 2H), 7.28 (d, *J* = 8.0 Hz, 2H), 7.13 (s, 2H), 5.72 (d, *J* = 2.5 Hz, 1H), 5.21 (s, 1H), 2.16 (d, *J* = 3.1 Hz, 1H), 1.41 (s, 18H); ^{13}C NMR (100 MHz, CDCl$_3$) δ 153.50, 143.07, 136.02, 134.09, 131.32, 128.06, 123.57, 120.97, 76.13, 34.37, 30.20.

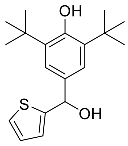

3t

2,6-di-*tert*-Butyl-4-(hydroxy(thiophen-2-yl)methyl)phenol 3t:[1] ^{1}H NMR (400 MHz, CDCl$_3$) δ 7.28 – 7.23 (m, 3H), 6.94 (dd, *J* = 5.1, 3.5 Hz, 1H), 6.91 – 6.83 (m, 1H), 5.97 (d, *J* = 2.9 Hz, 1H), 5.23 (s, 1H), 2.37 (d, *J* = 3.6 Hz, 1H), 1.43 (s, 18H); ^{13}C NMR (100 MHz, CDCl$_3$) δ 153.68, 148.62, 135.94, 133.82, 126.58, 125.06, 124.65, 123.44, 73.18, 34.43, 30.29.

References

[1] Lou, Y.; Cao, P.; Jia, T.; Zhang, Y.; Wang, M.; Liao, J. *Angew. Chem., Int. Ed.* **2015**, *54*, 12134.

[2] Li, W.; Wen, W.; Chen, S.; Ding, L.; He, B.; Zhang, Y.; Li, B.; Zhu, L. *Cat. Lett.* **2022**, DOI: 10.1007/s10562-022-04063-7.

NMR Spectral of Products

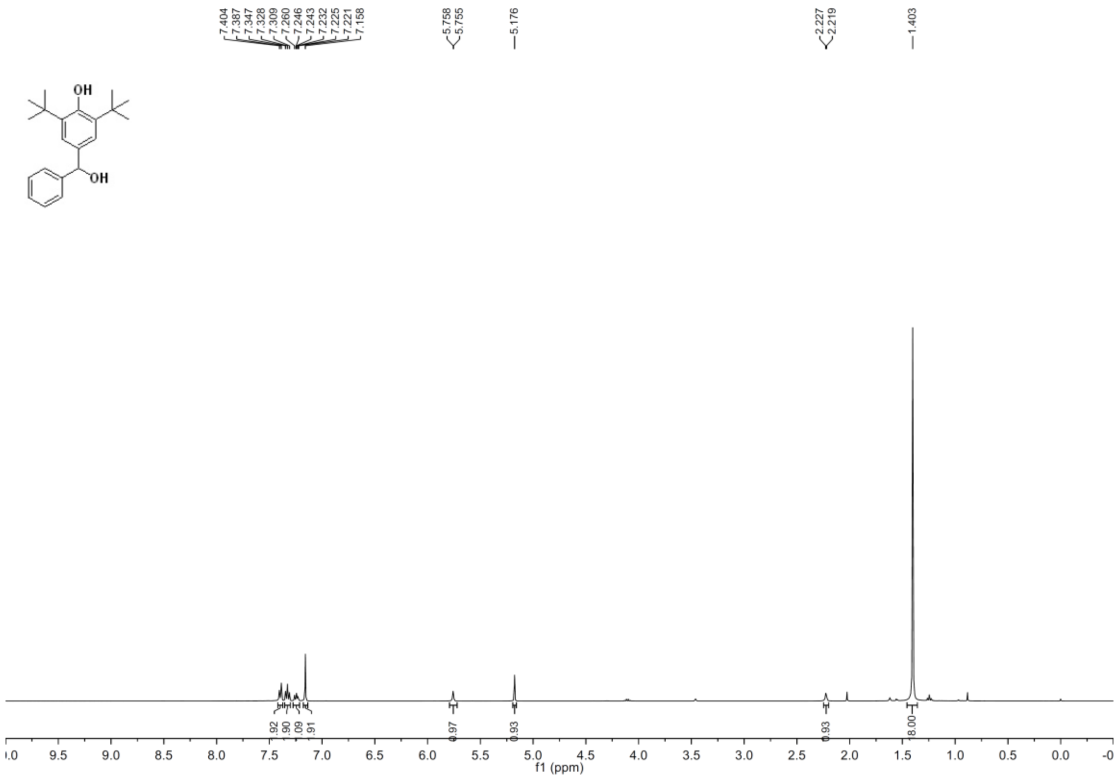

^{1}H NMR (400 MHz, $CDCl_3$) of compound **3a**

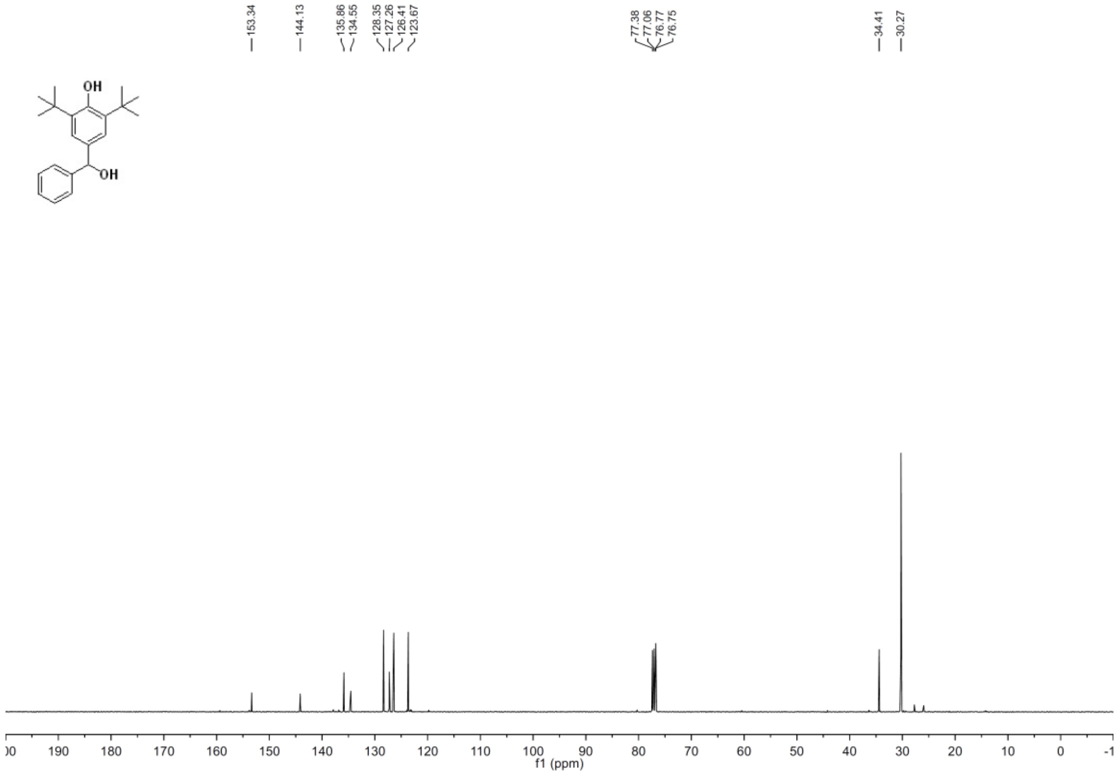

^{13}C NMR (100 MHz, $CDCl_3$) of compound **3a**

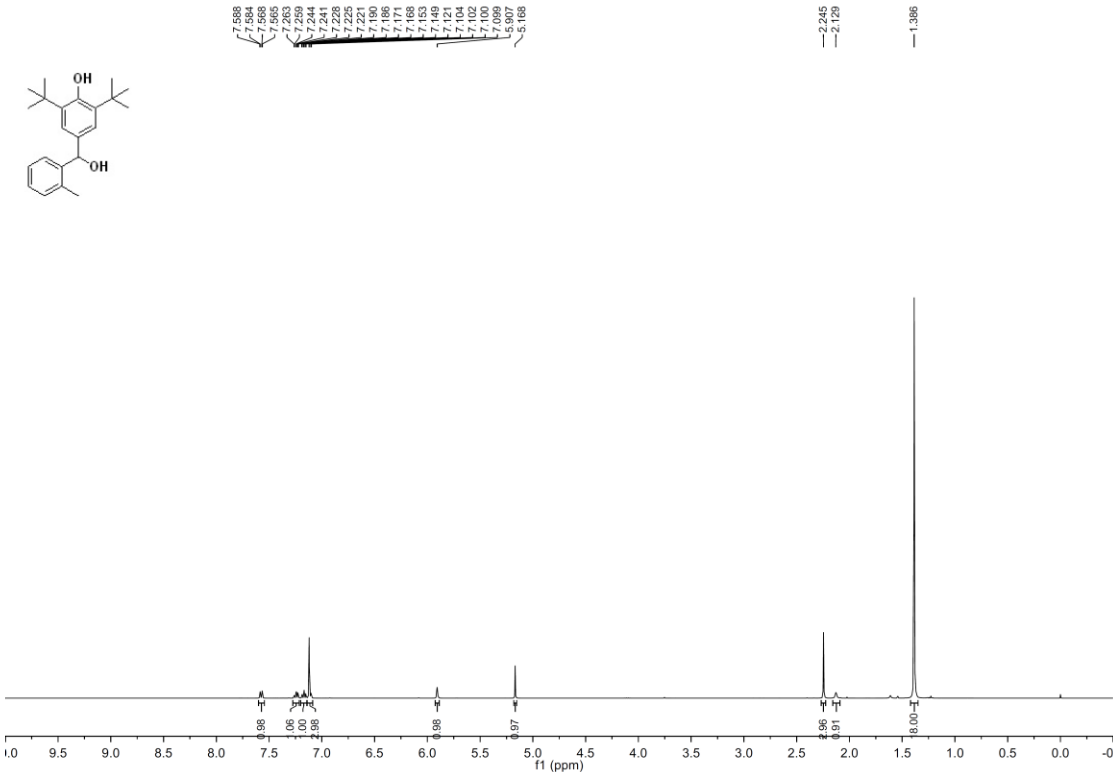

^{1}H NMR (400 MHz, $CDCl_3$) of compound **3b**

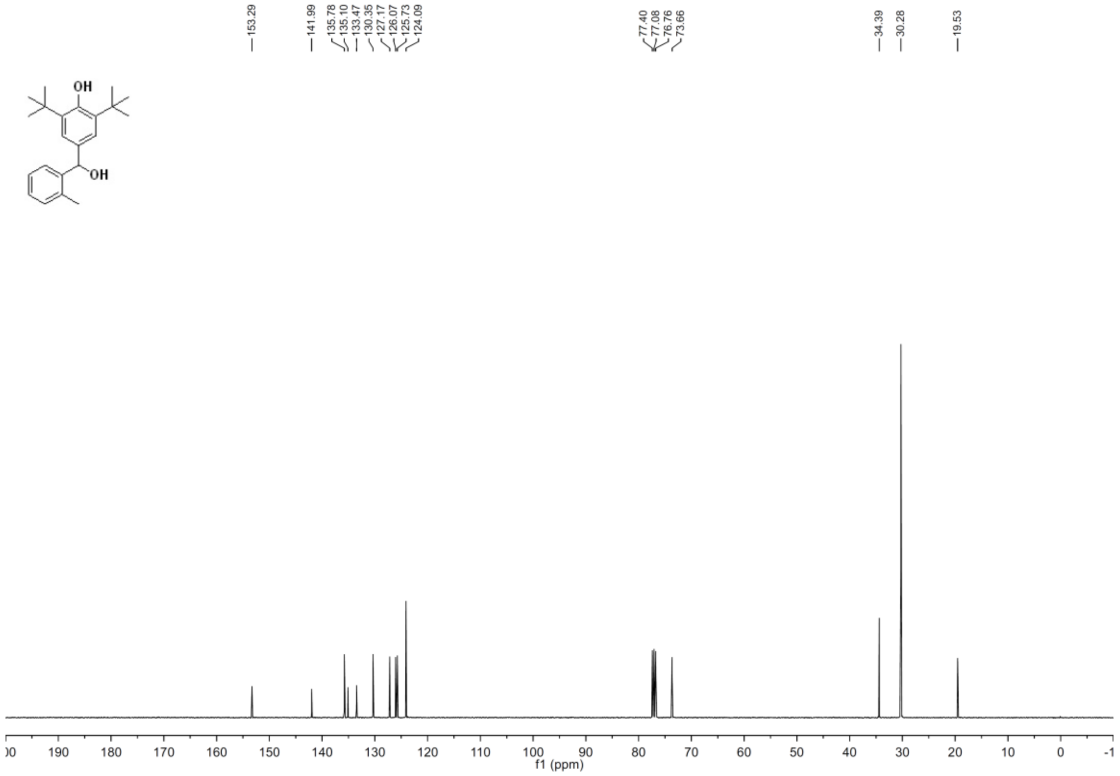

^{13}C NMR (100 MHz, $CDCl_3$) of compound **3b**

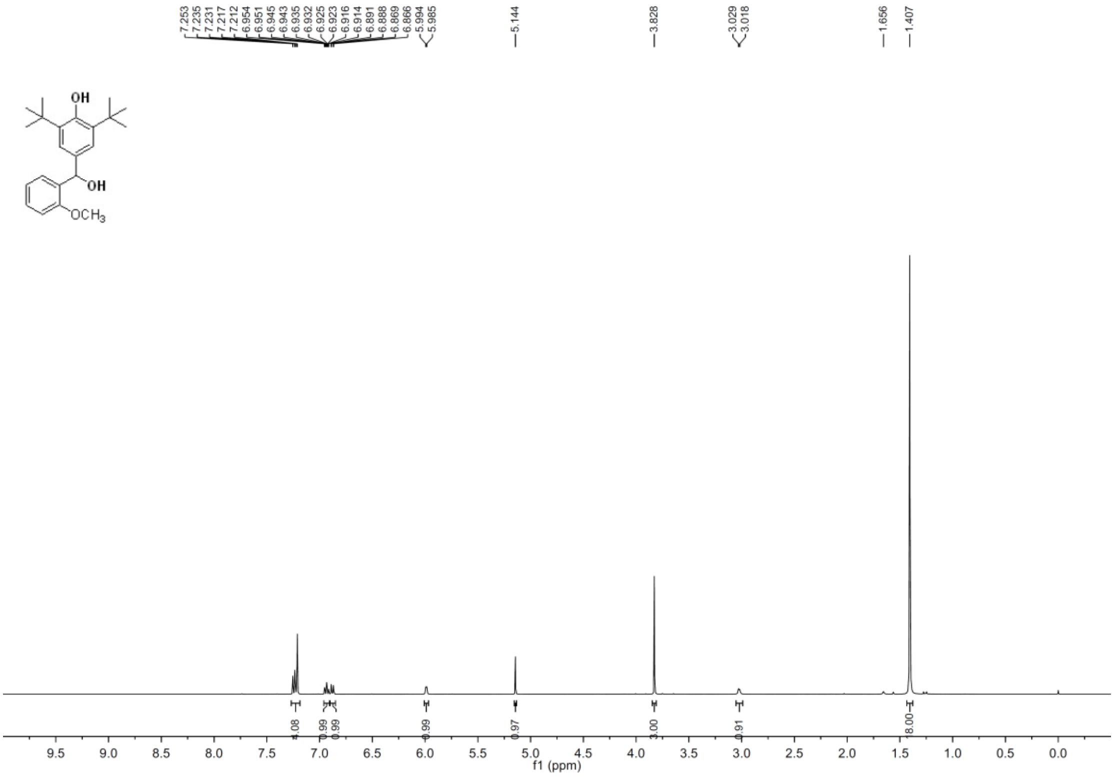

^{1}H NMR (400 MHz, $CDCl_3$) of compound **3c**

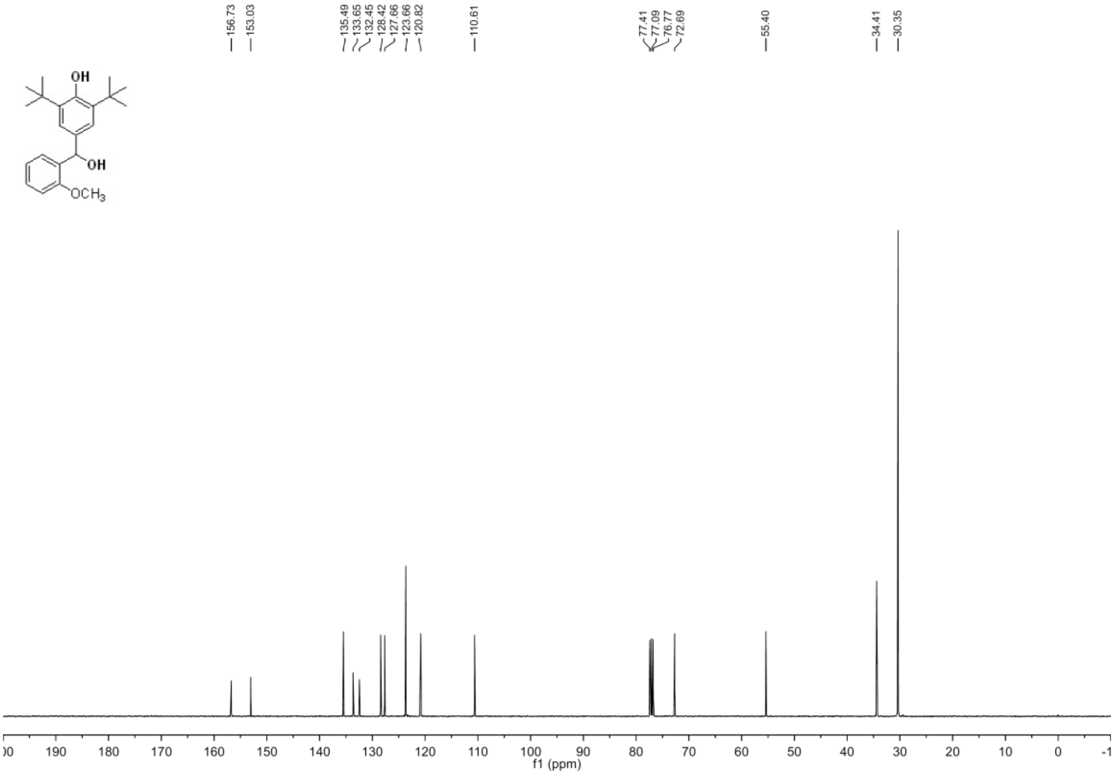

^{13}C NMR (100 MHz, $CDCl_3$) of compound **3c**

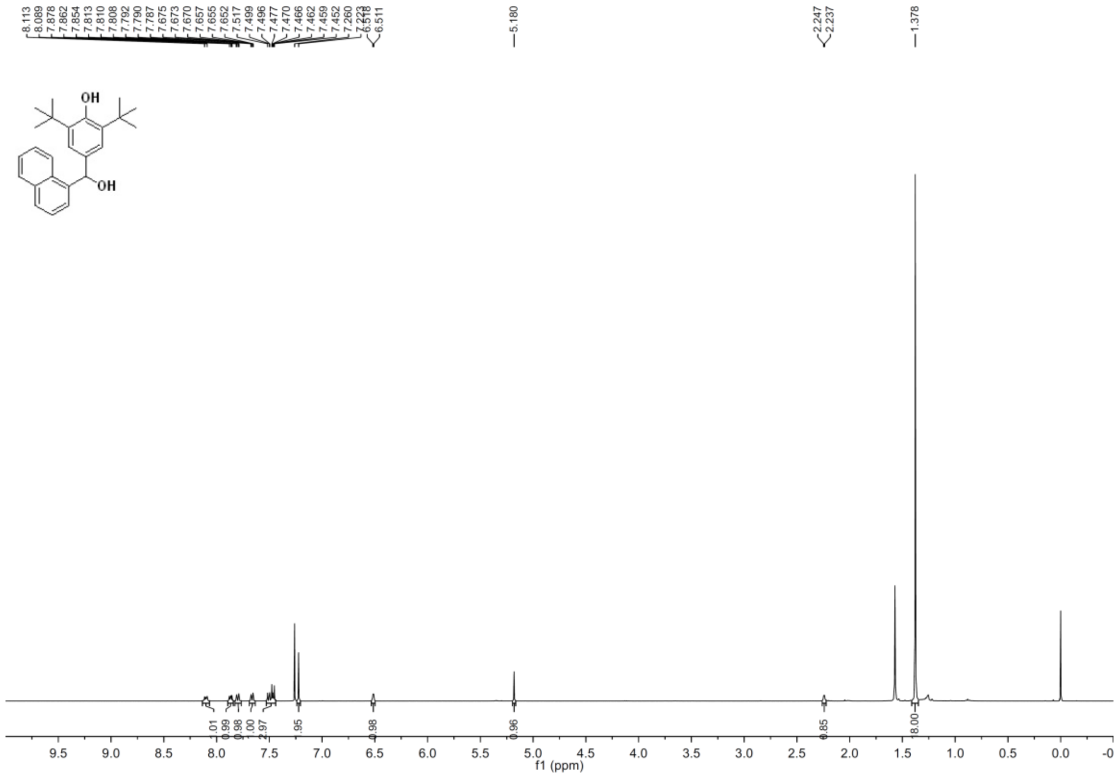

^{1}H NMR (400 MHz, $CDCl_3$) of compound **3d**

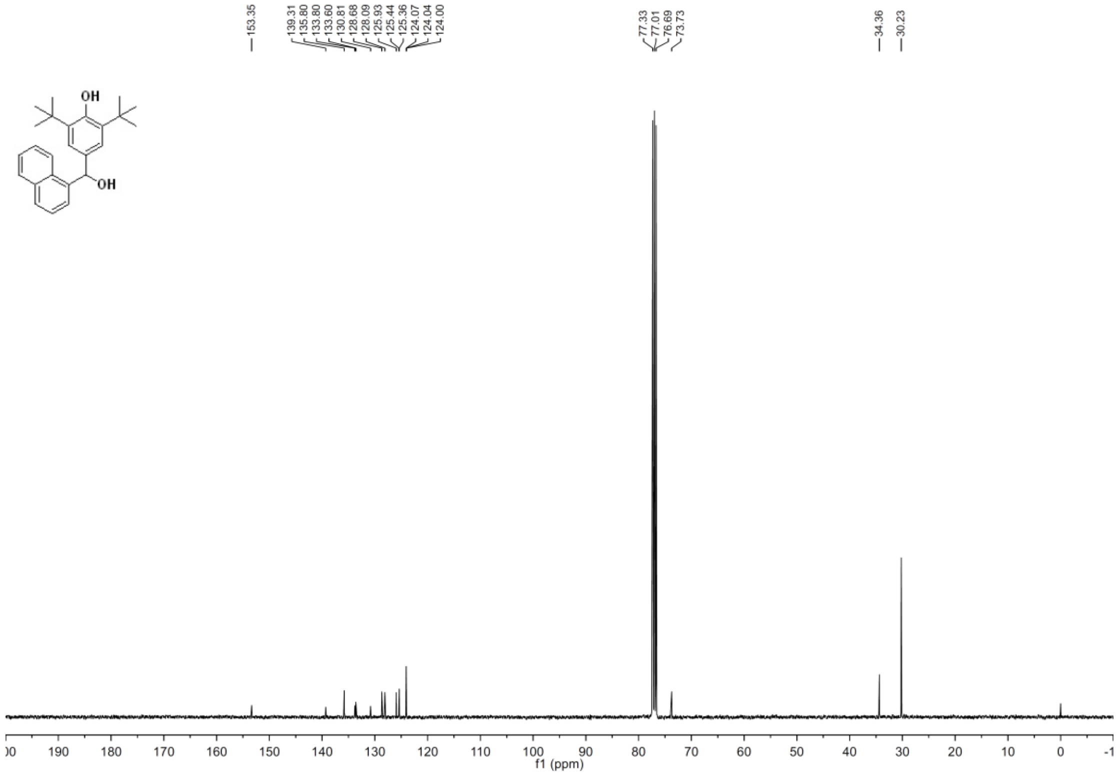

^{13}C NMR (100 MHz, $CDCl_3$) of compound **3d**

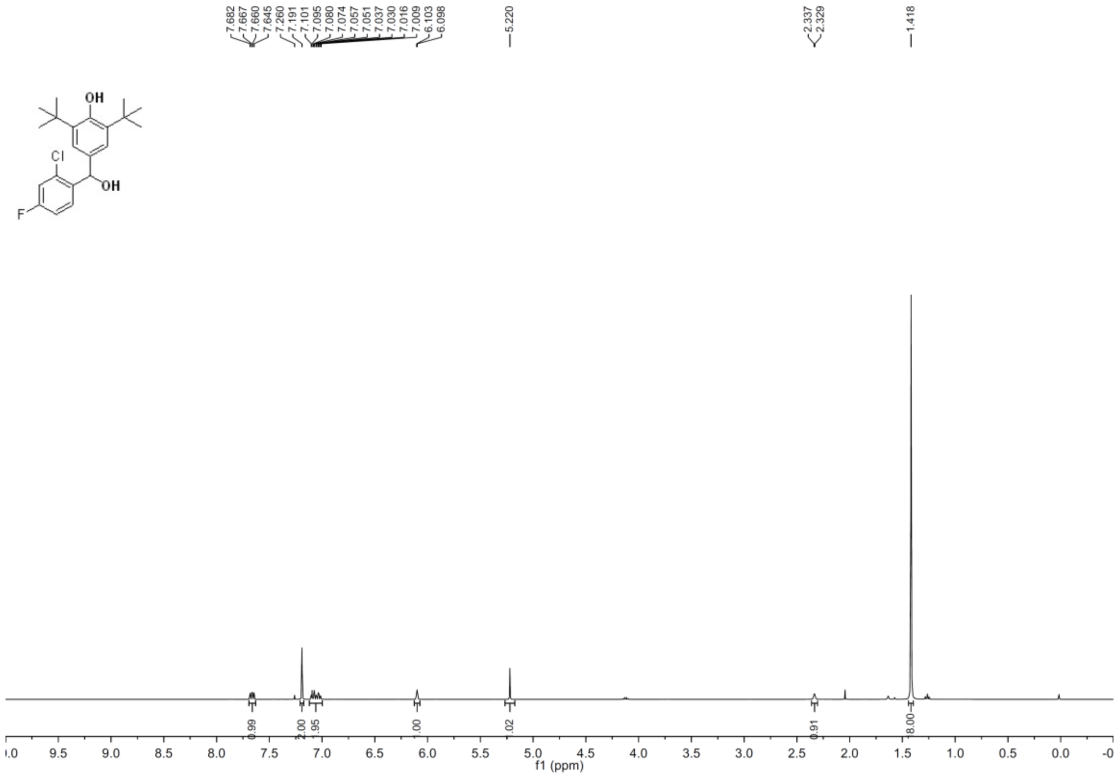

^{1}H NMR (400 MHz, $CDCl_3$) of compound **3e**

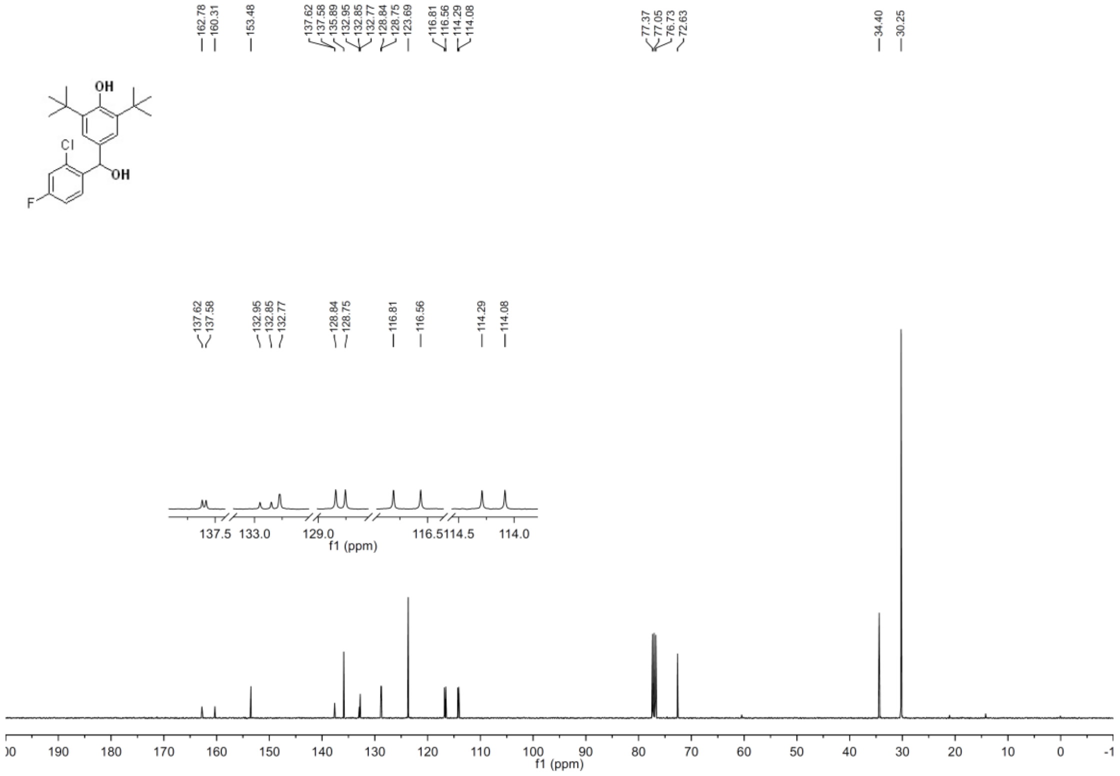

^{13}C NMR (100 MHz, $CDCl_3$) of compound **3e**

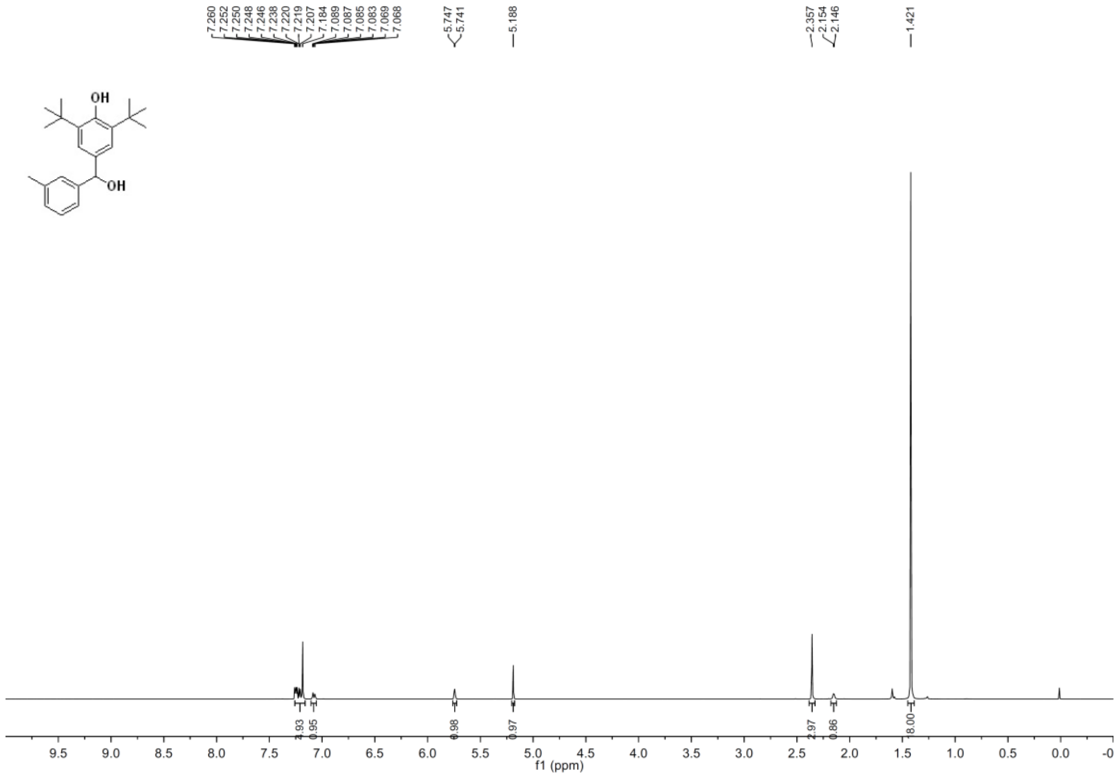

^{1}H NMR (400 MHz, $CDCl_3$) of compound **3f**

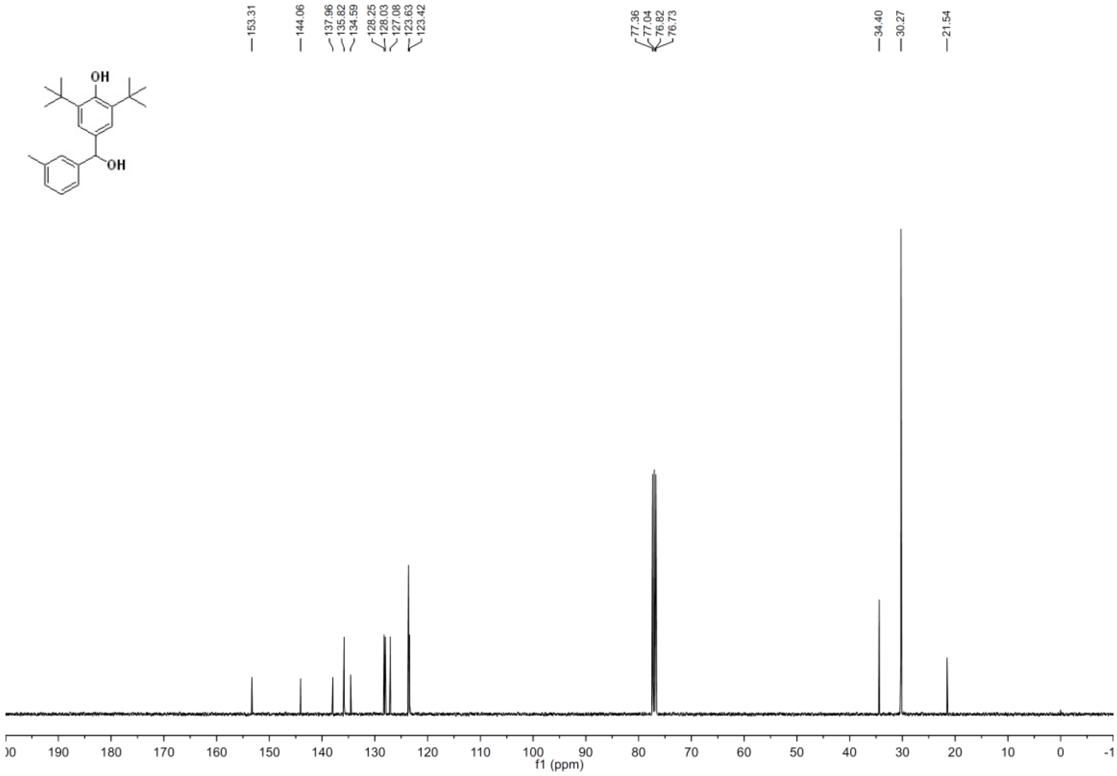

^{13}C NMR (100 MHz, $CDCl_3$) of compound **3f**

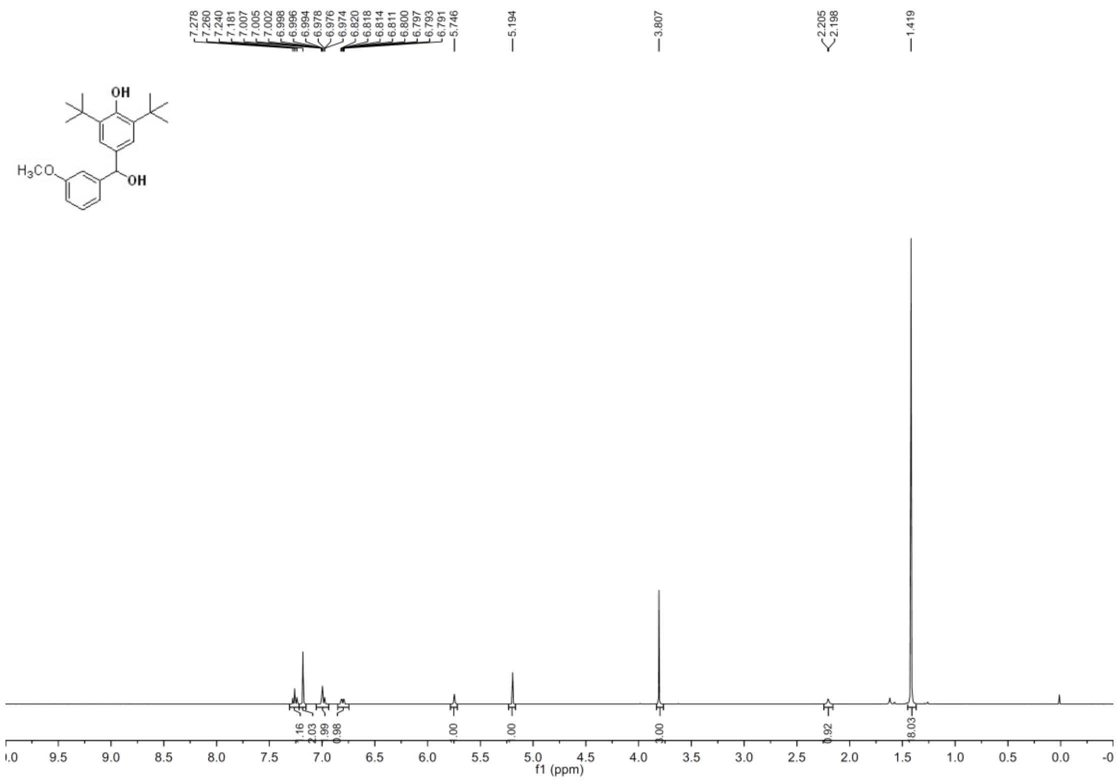

^{1}H NMR (400 MHz, CDCl3) of compound **3g**

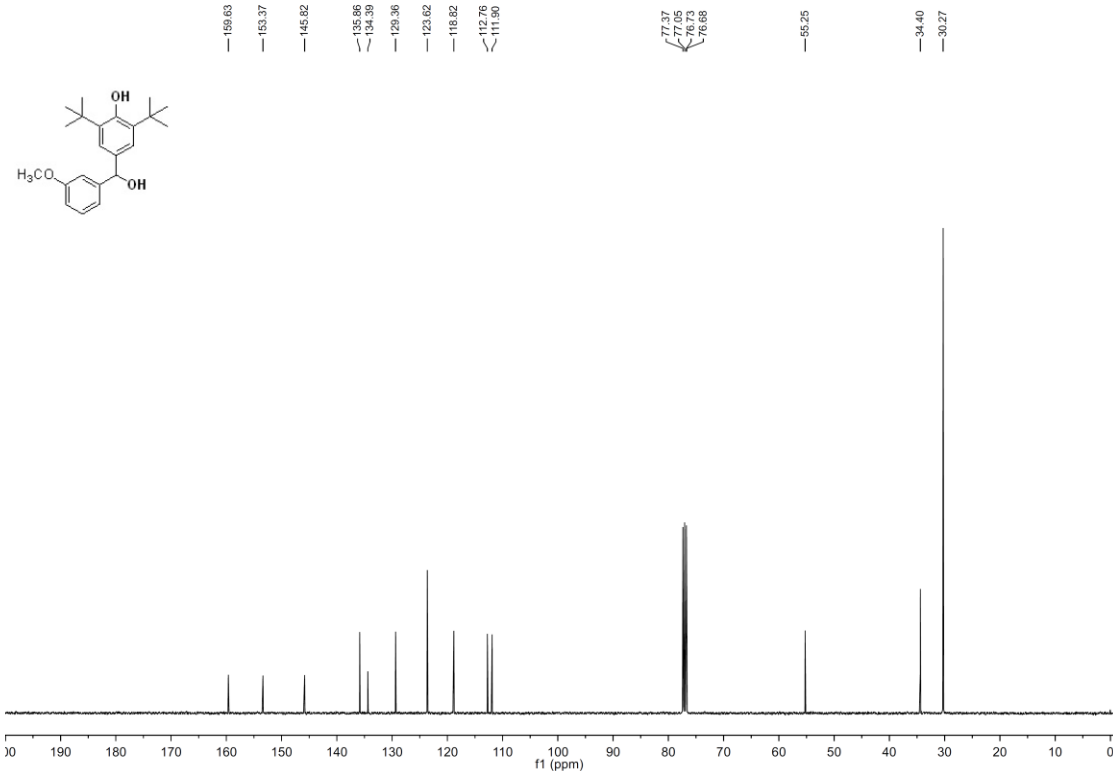

^{13}C NMR (100 MHz, CDCl3) of compound **3g**

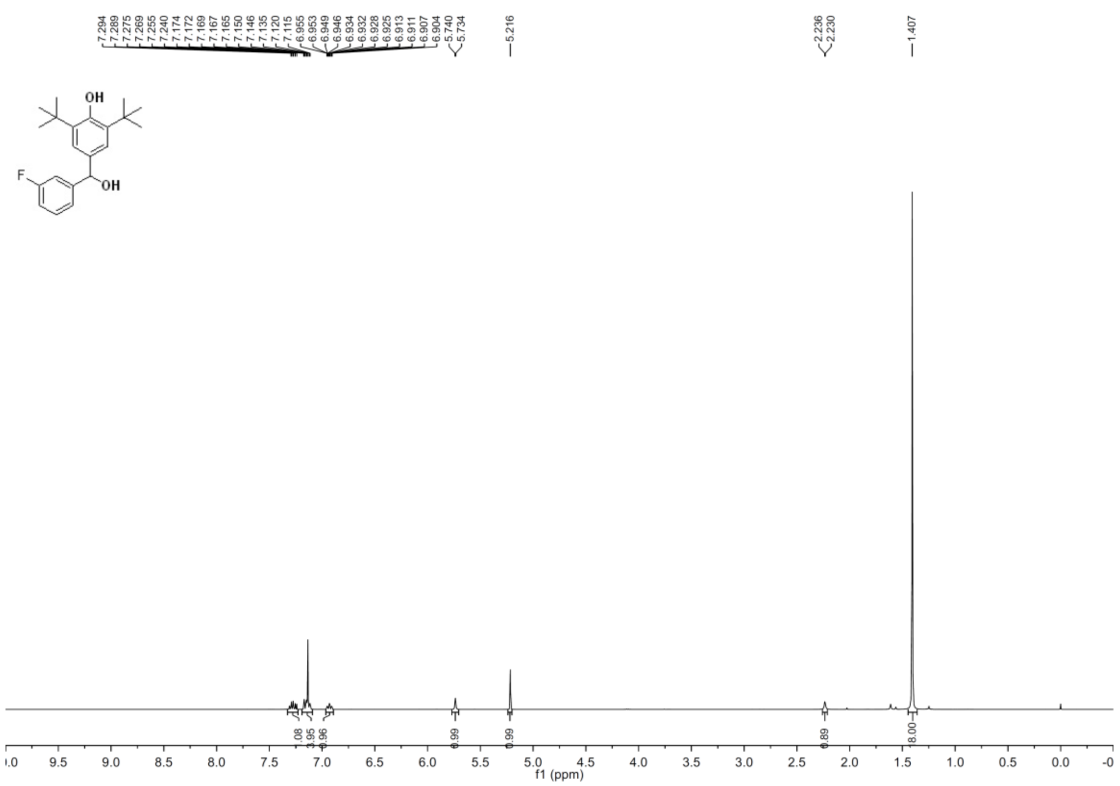

^{1}H NMR (400 MHz, $CDCl_3$) of compound **3h**

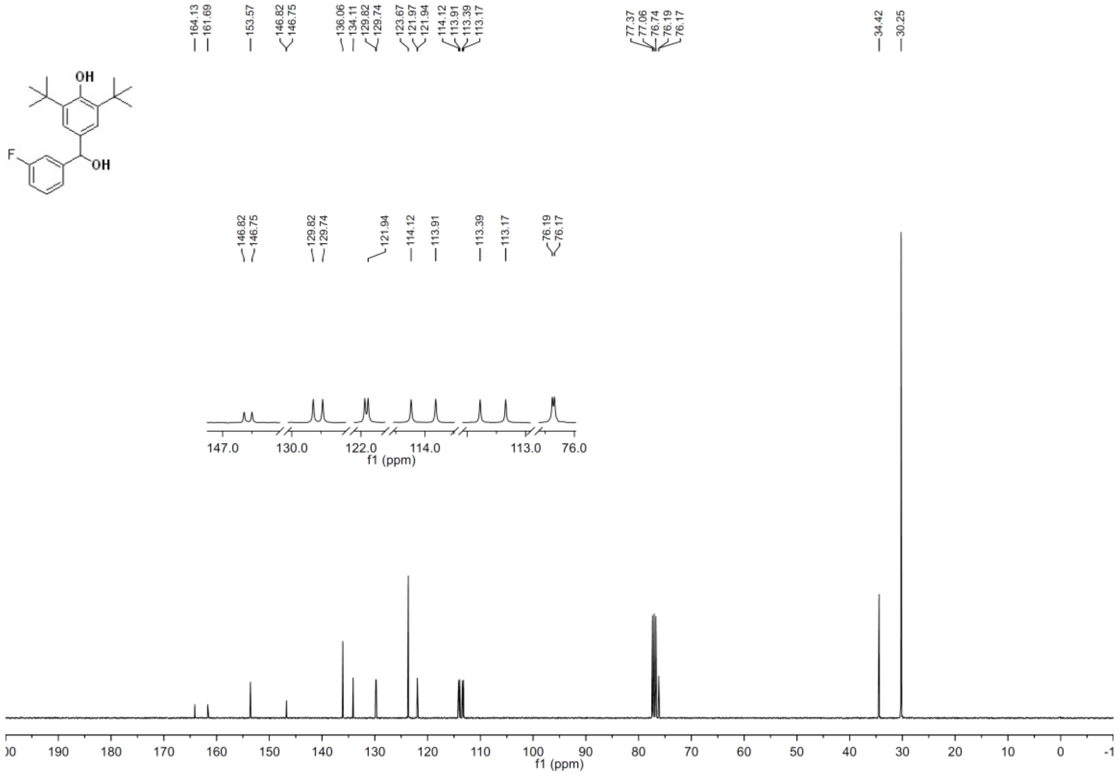

^{13}C NMR (100 MHz, $CDCl_3$) of compound **3h**

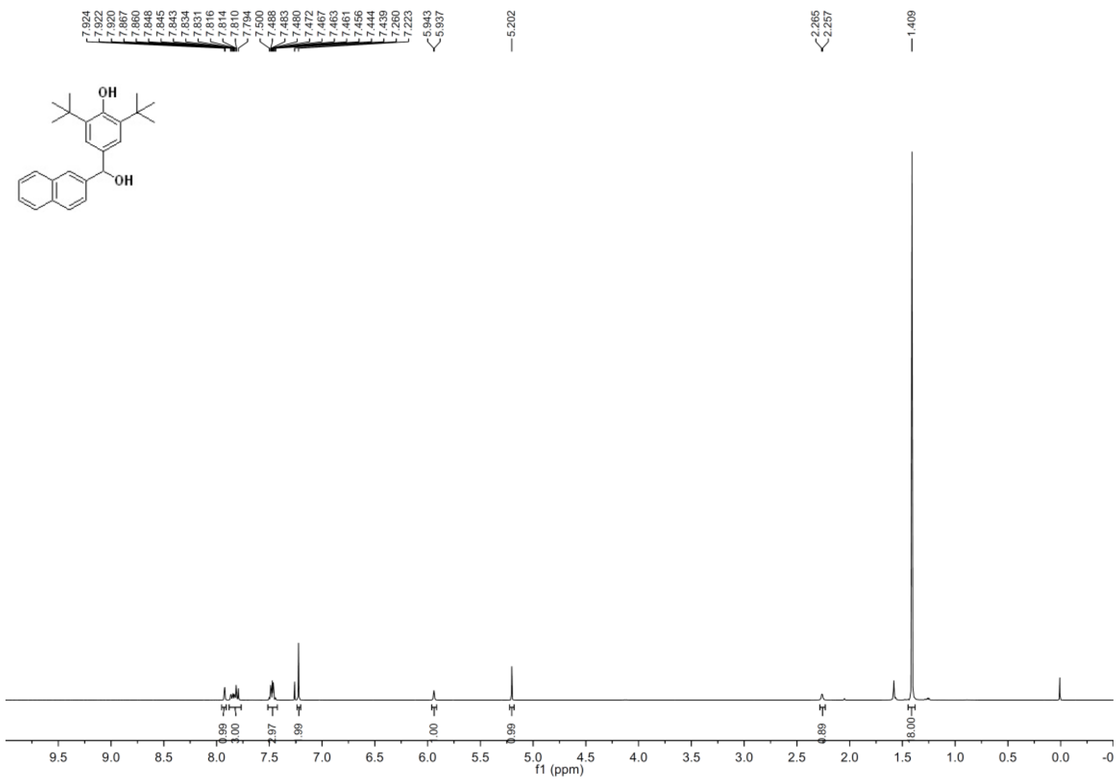

^{1}H NMR (400 MHz, $CDCl_3$) of compound **3i**

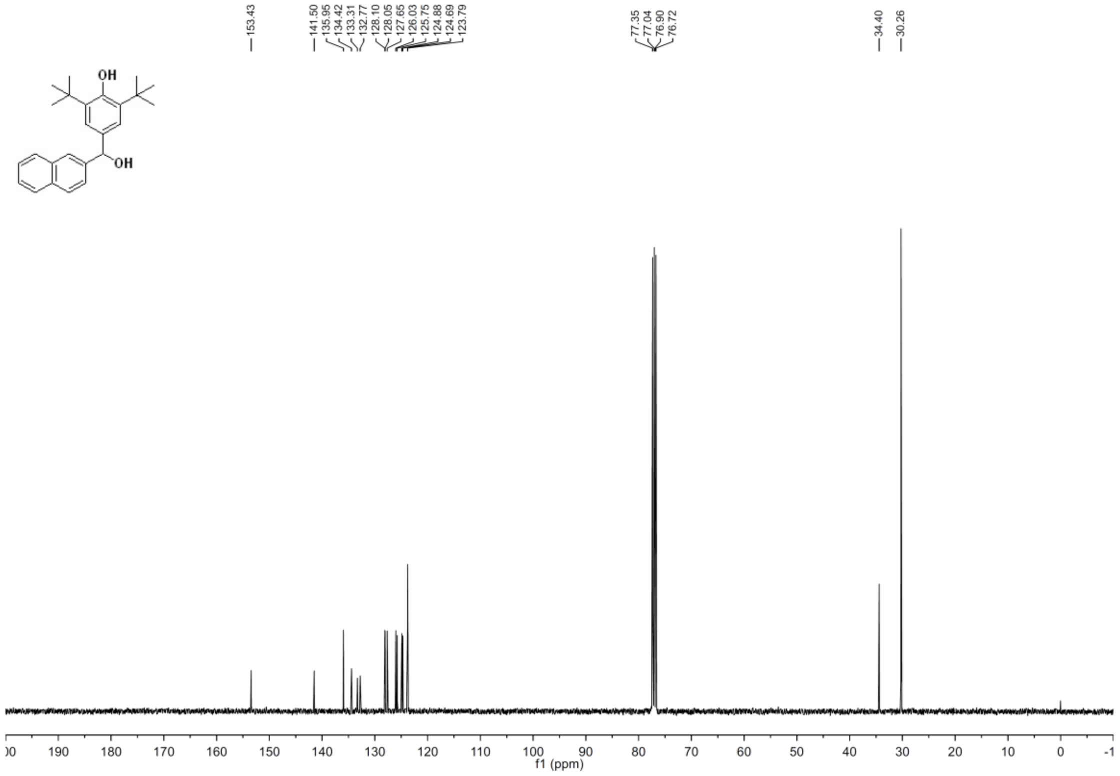

^{13}C NMR (100 MHz, $CDCl_3$) of compound **3i**

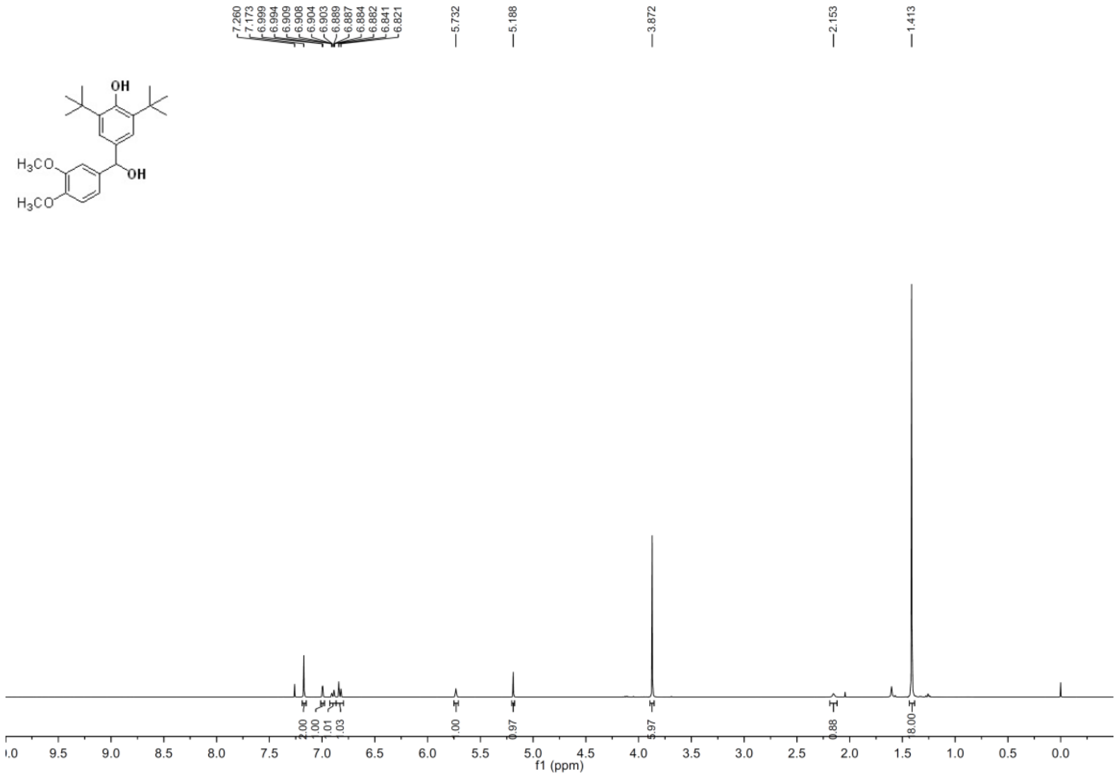

^{1}H NMR (400 MHz, $CDCl_3$) of compound **3j**

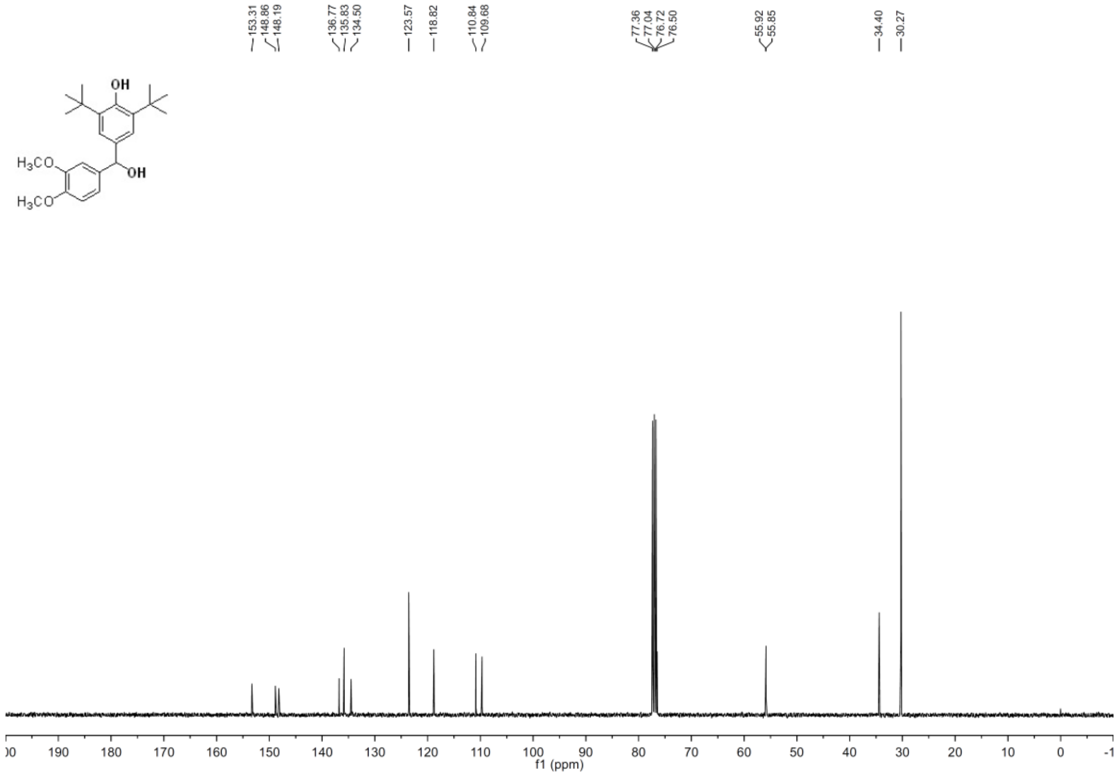

^{13}C NMR (100 MHz, $CDCl_3$) of compound **3j**

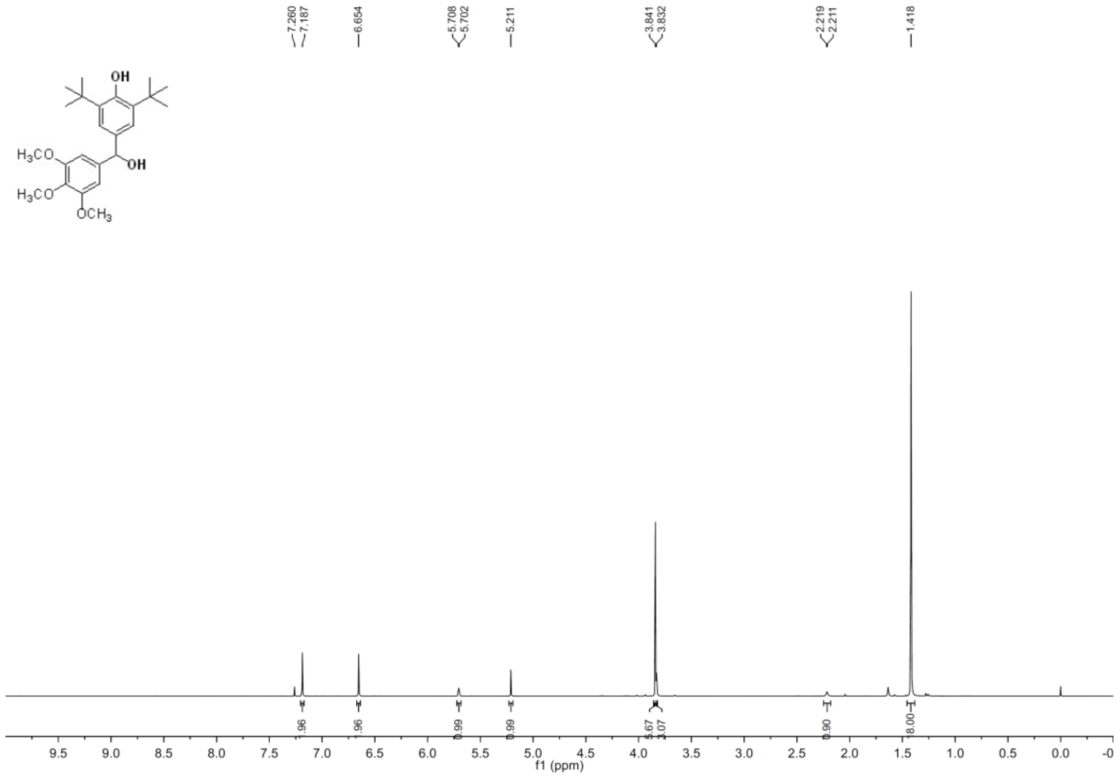

^{1}H NMR (400 MHz, $CDCl_3$) of compound **3k**

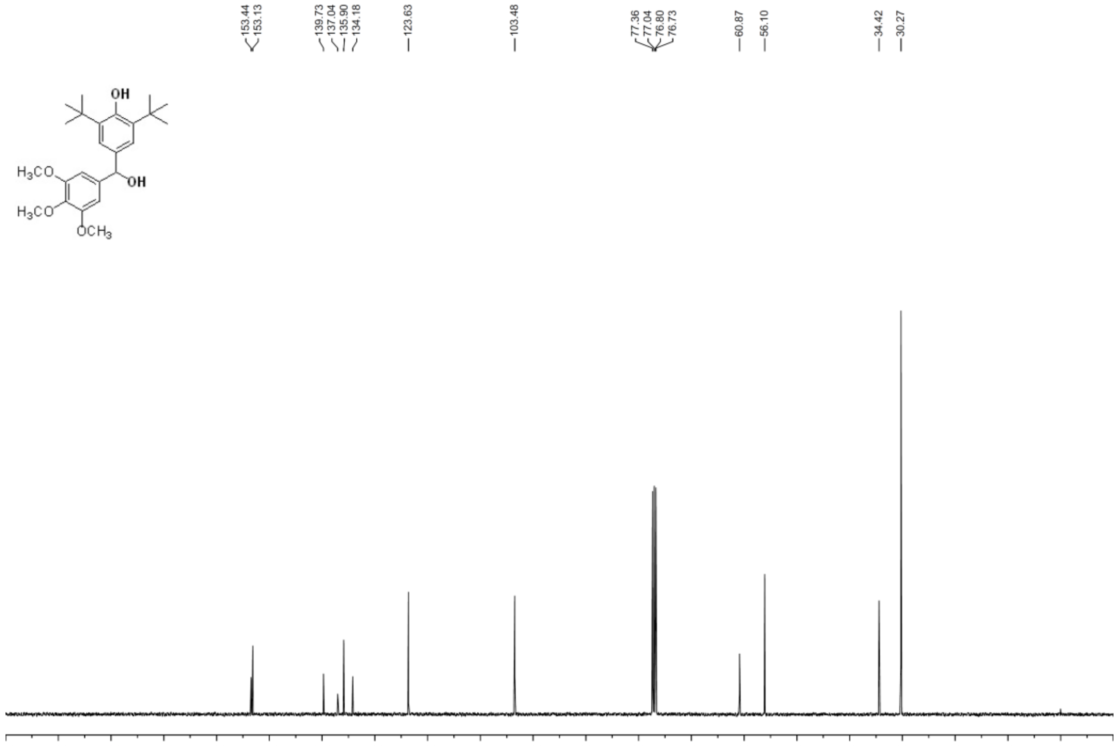

^{13}C NMR (100 MHz, $CDCl_3$) of compound **3k**

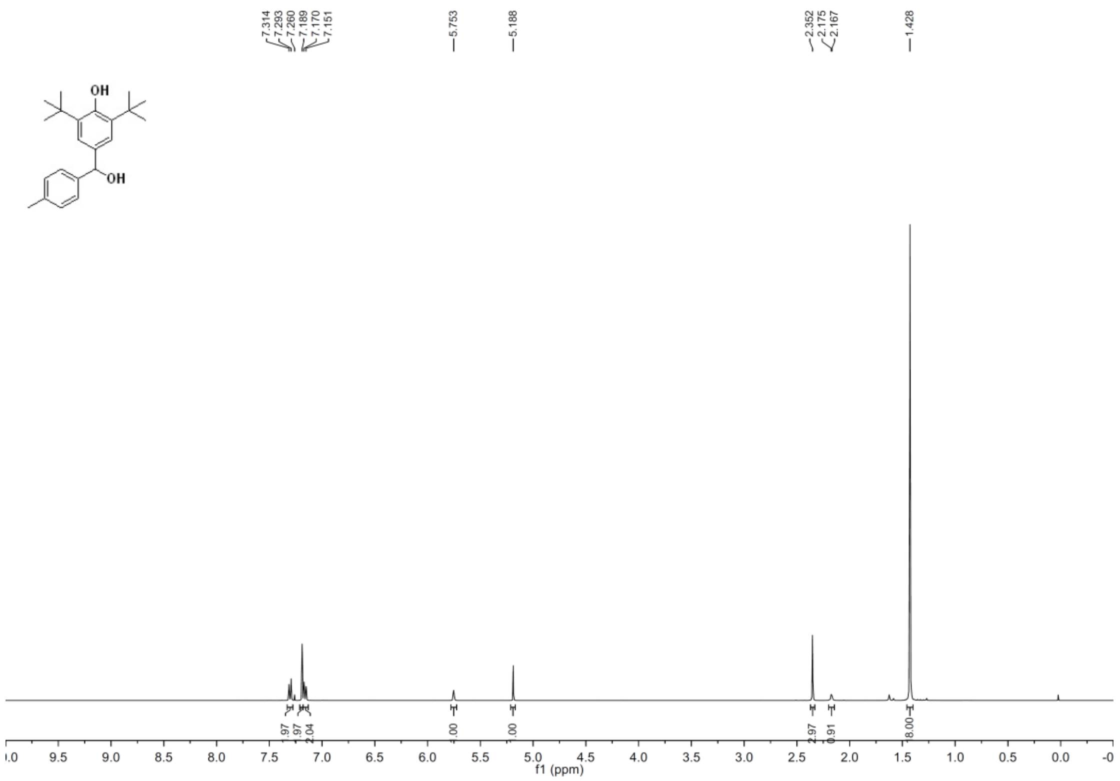

^{1}H NMR (400 MHz, $CDCl_3$) of compound **3l**

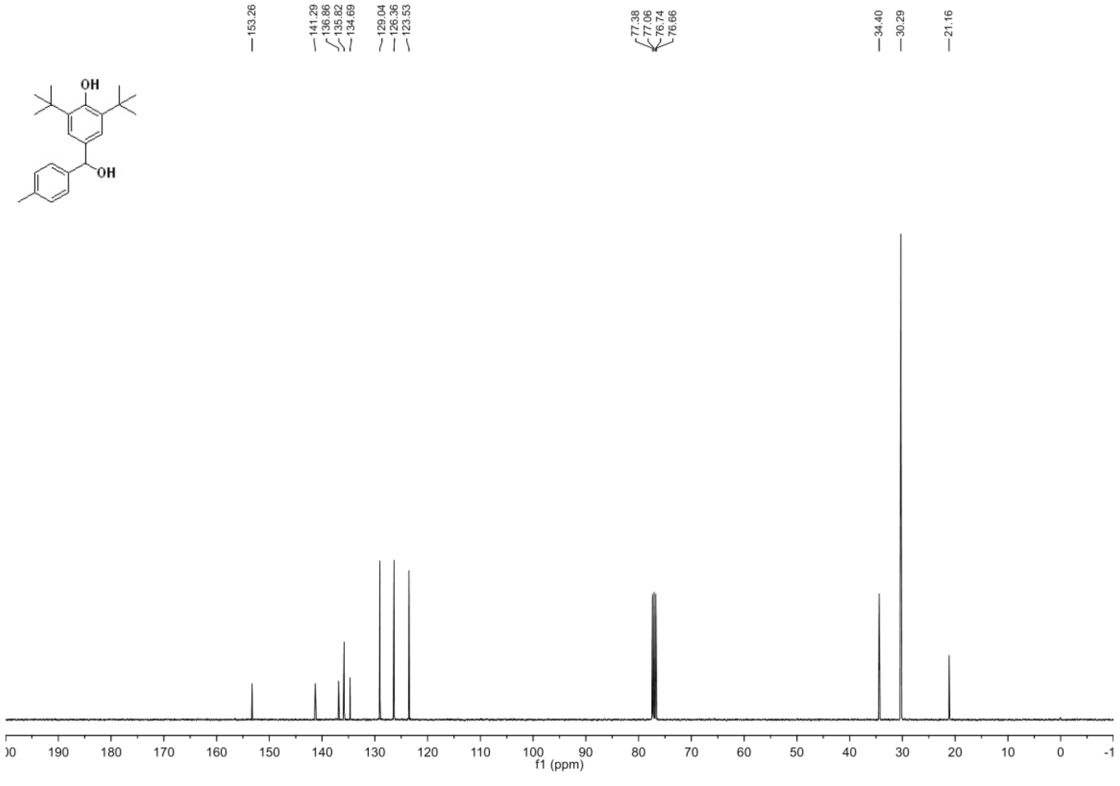

^{13}C NMR (100 MHz, $CDCl_3$) of compound **3l**

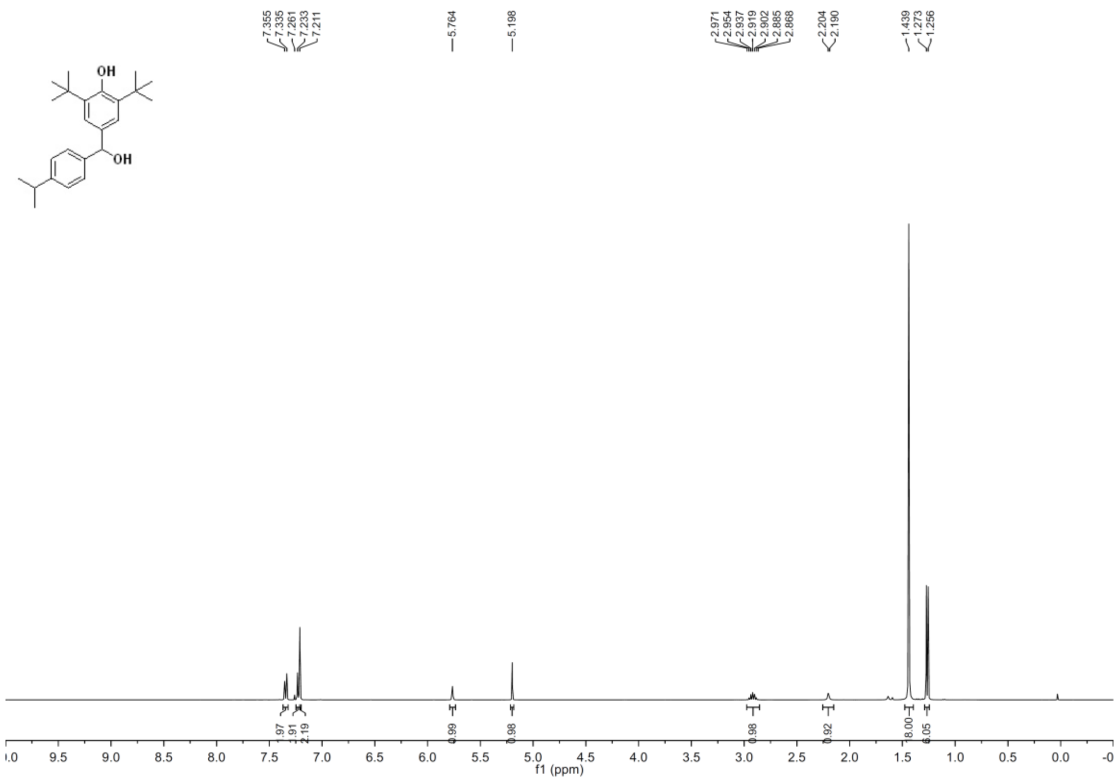

^{1}H NMR (400 MHz, $CDCl_3$) of compound **3m**

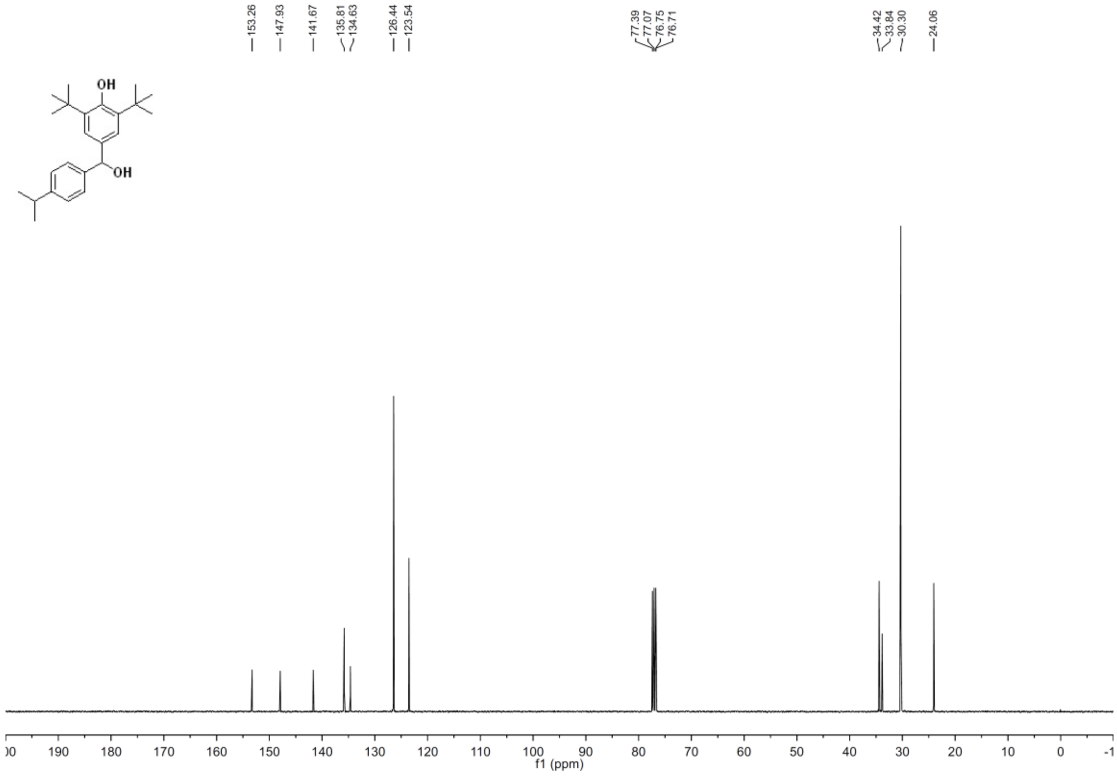

^{13}C NMR (100 MHz, $CDCl_3$) of compound **3m**

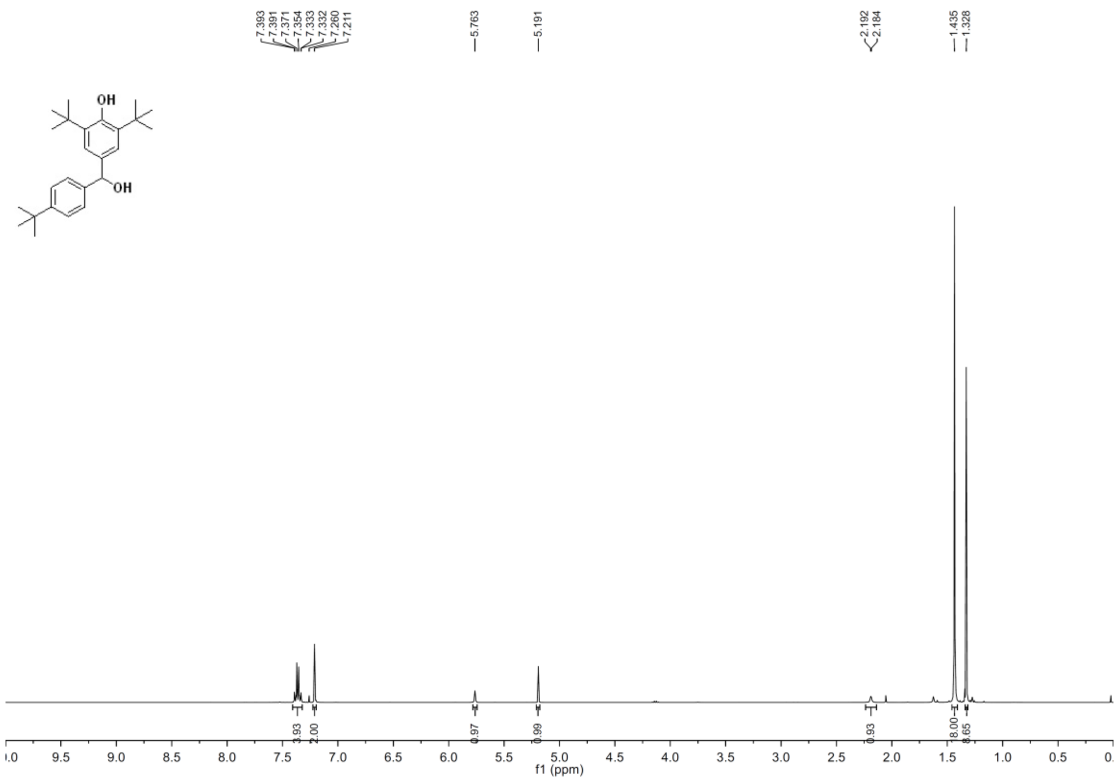

^{1}H NMR (400 MHz, $CDCl_3$) of compound **3n**

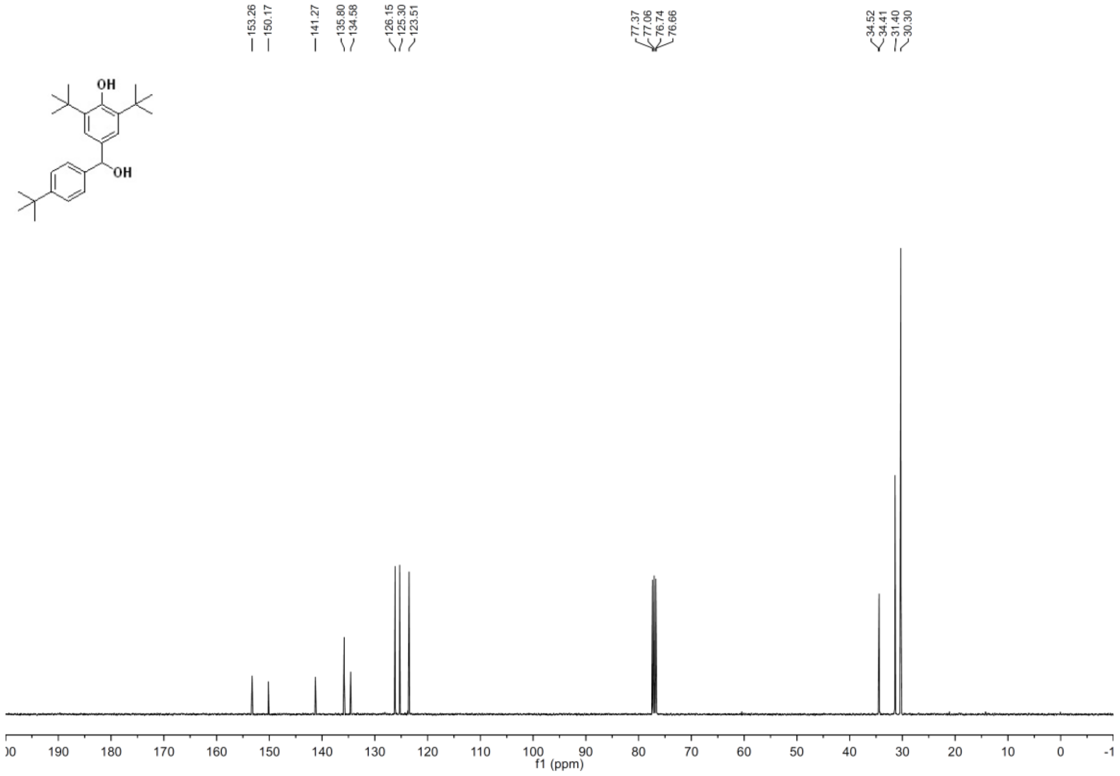

^{13}C NMR (100 MHz, $CDCl_3$) of compound **3n**

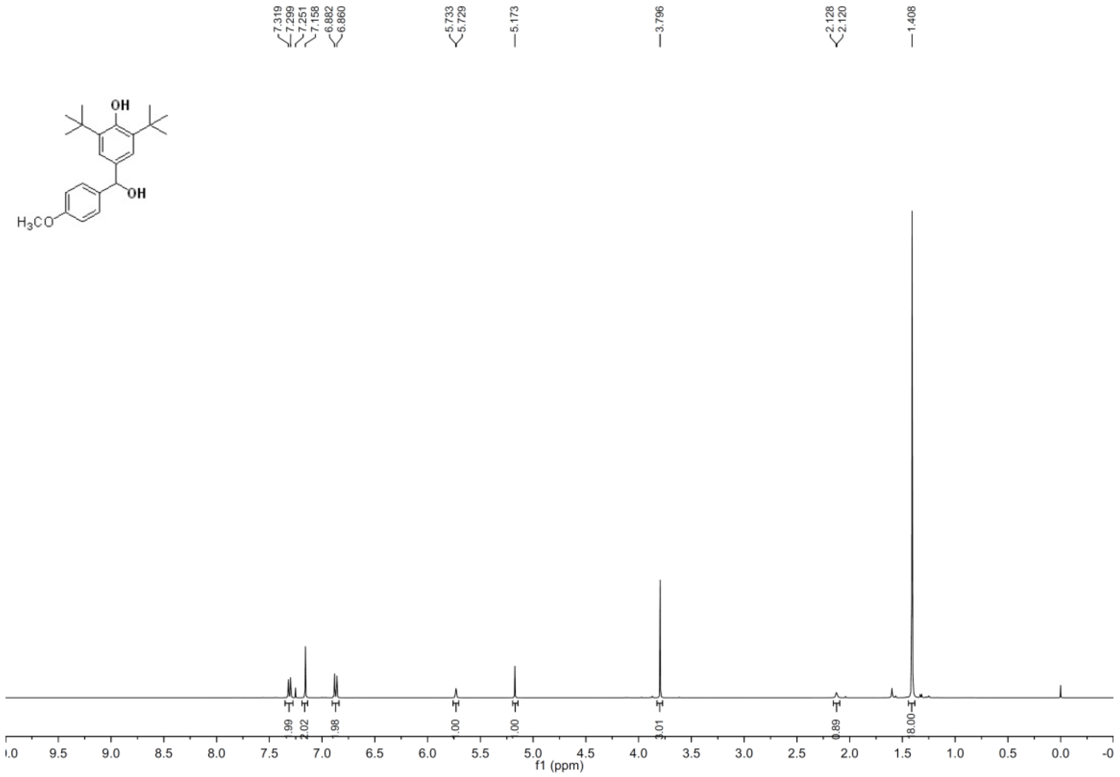

^{1}H NMR (400 MHz, $CDCl_3$) of compound **3o**

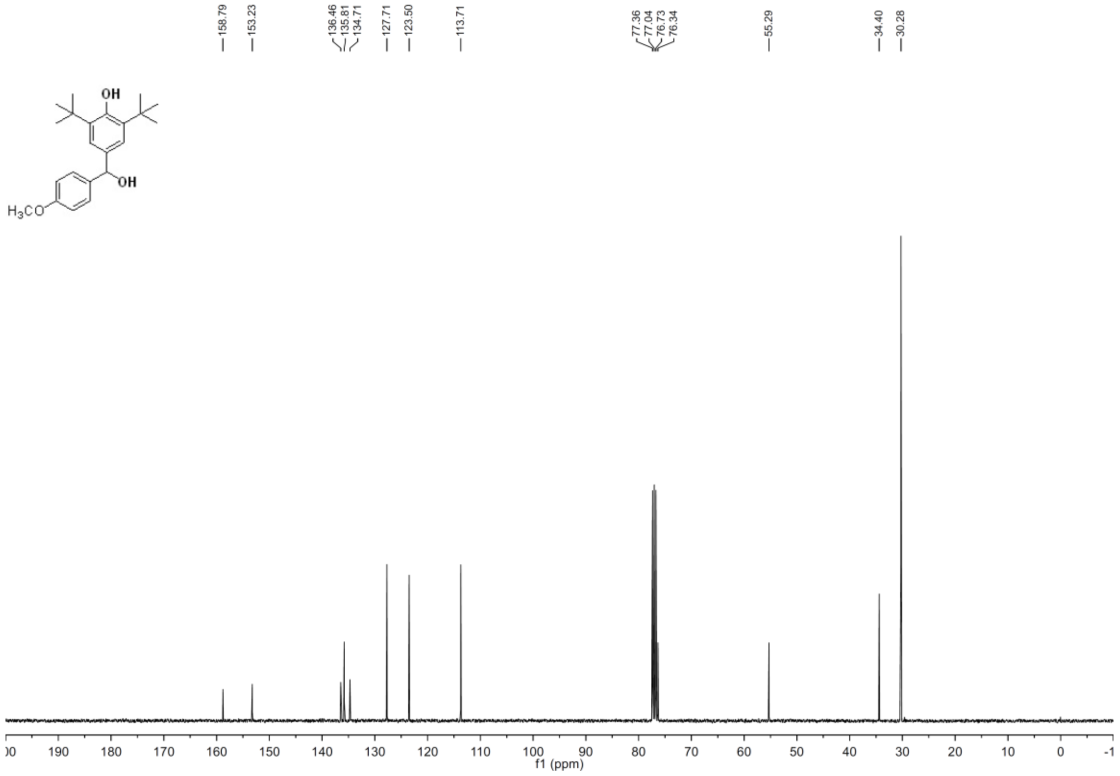

^{13}C NMR (100 MHz, $CDCl_3$) of compound **3o**

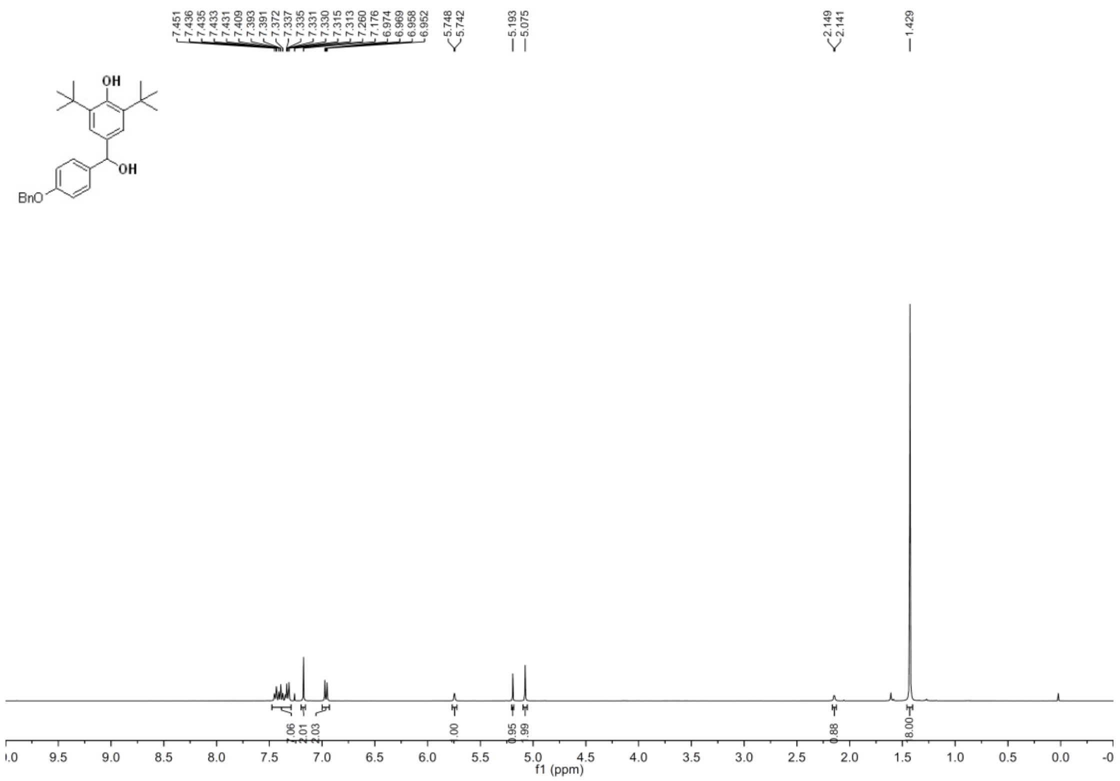

^{1}H NMR (400 MHz, $CDCl_3$) of compound **3p**

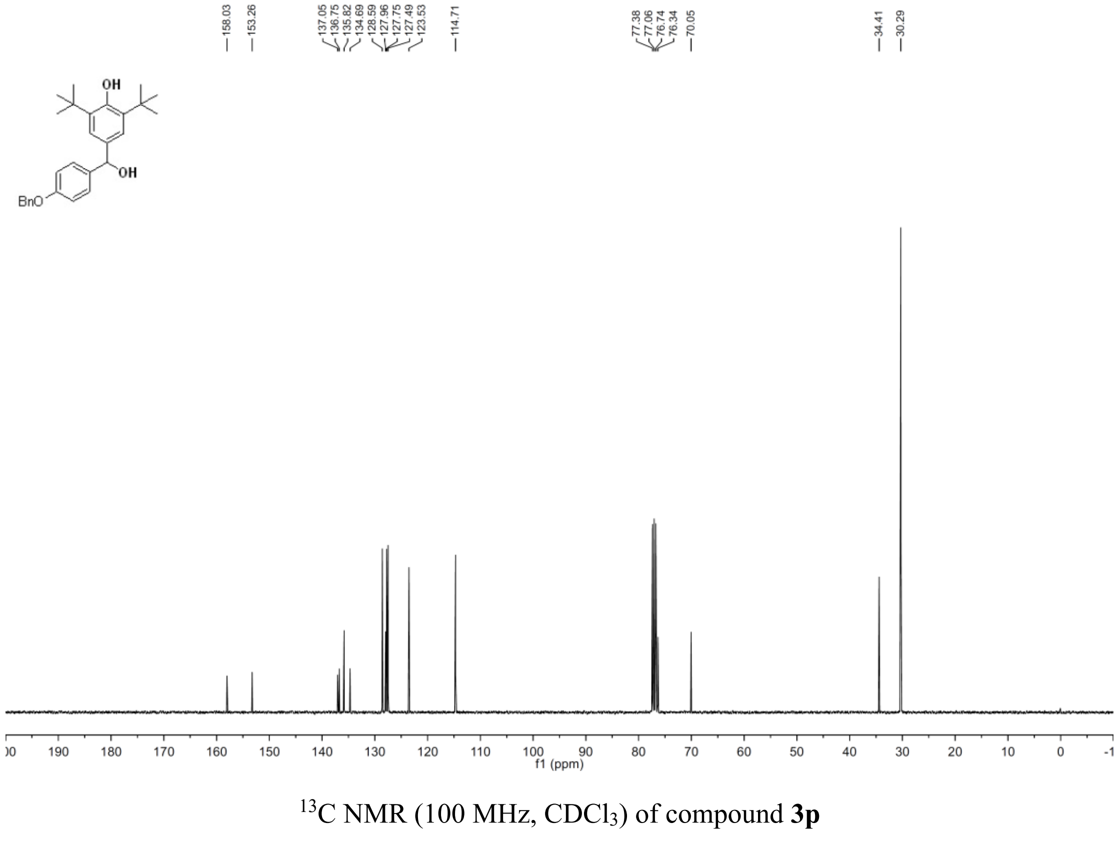

^{13}C NMR (100 MHz, $CDCl_3$) of compound **3p**

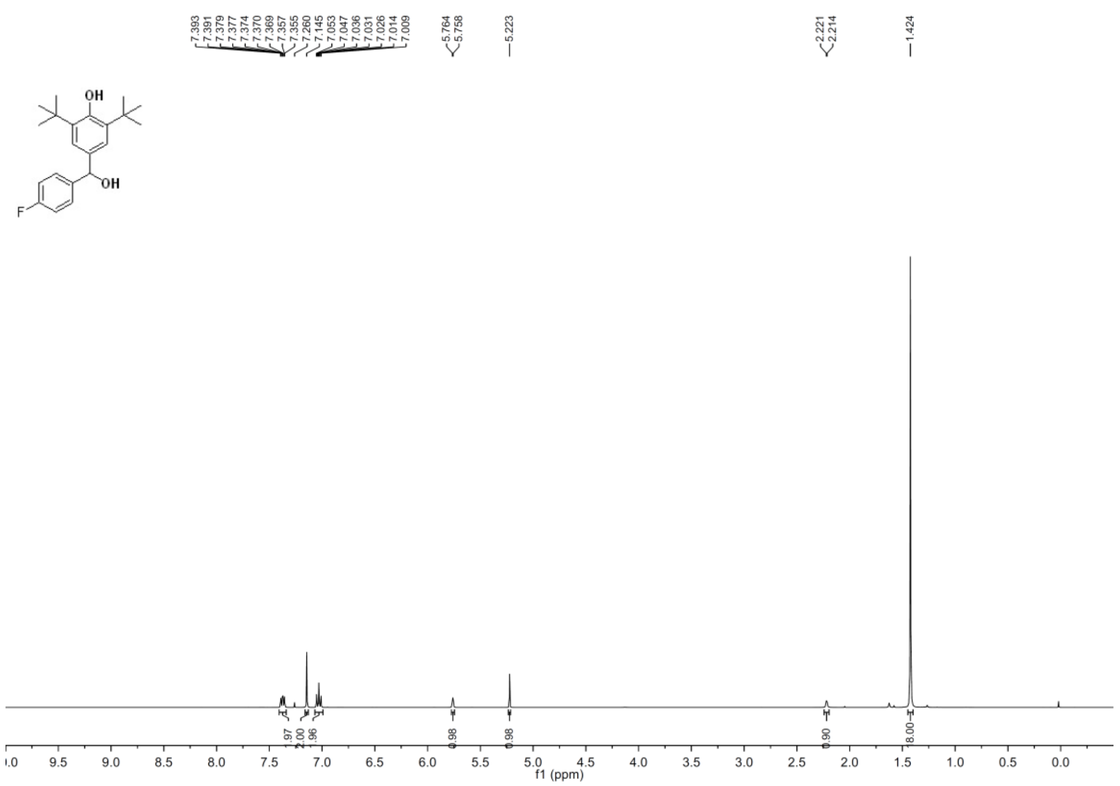

^{1}H NMR (400 MHz, $CDCl_3$) of compound **3q**

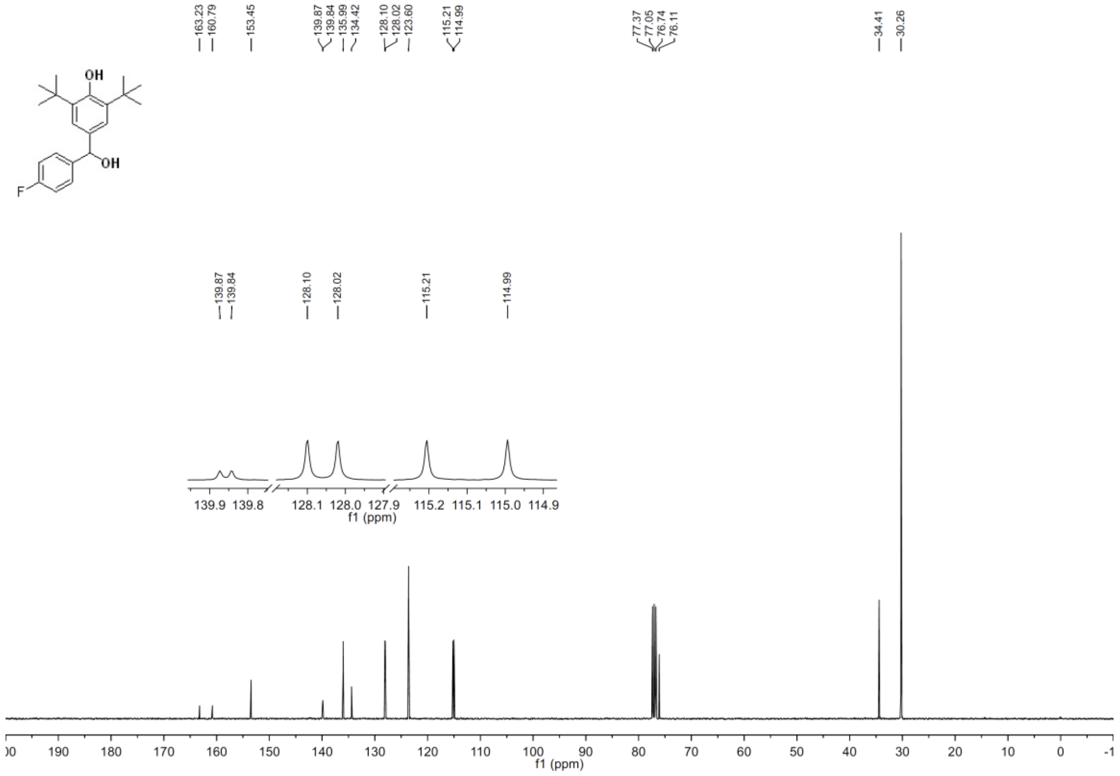

^{13}C NMR (100 MHz, $CDCl_3$) of compound **3q**

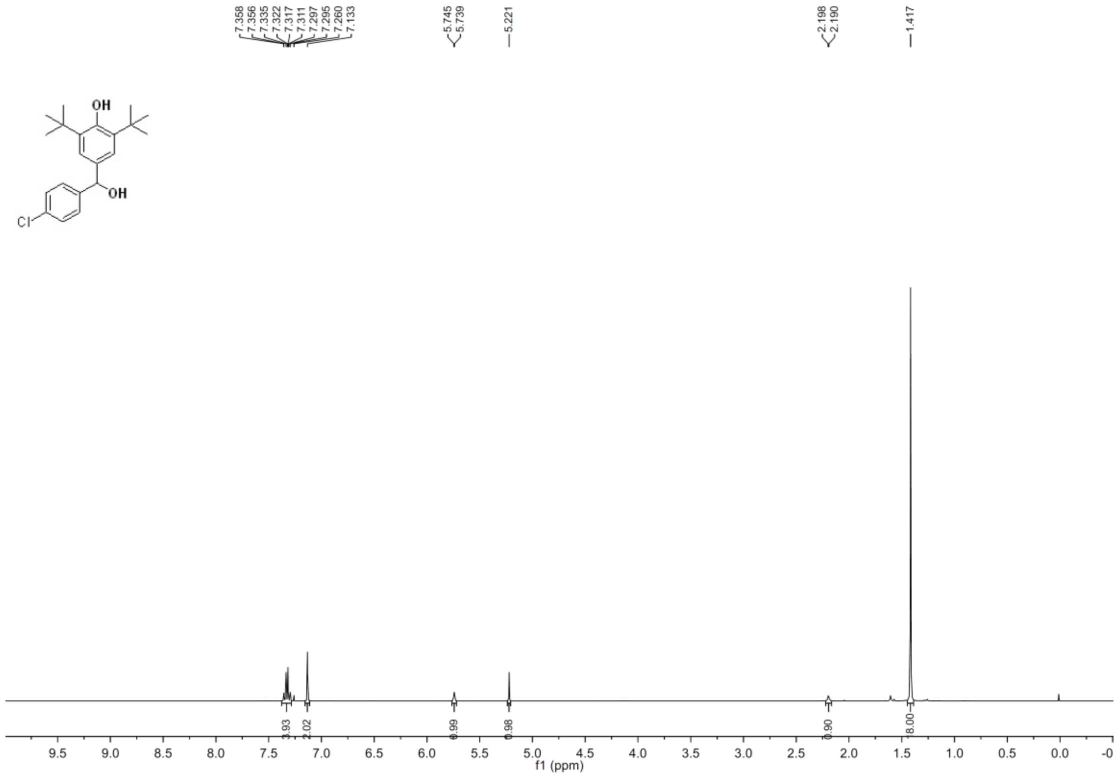

^{1}H NMR (400 MHz, $CDCl_3$) of compound **3r**

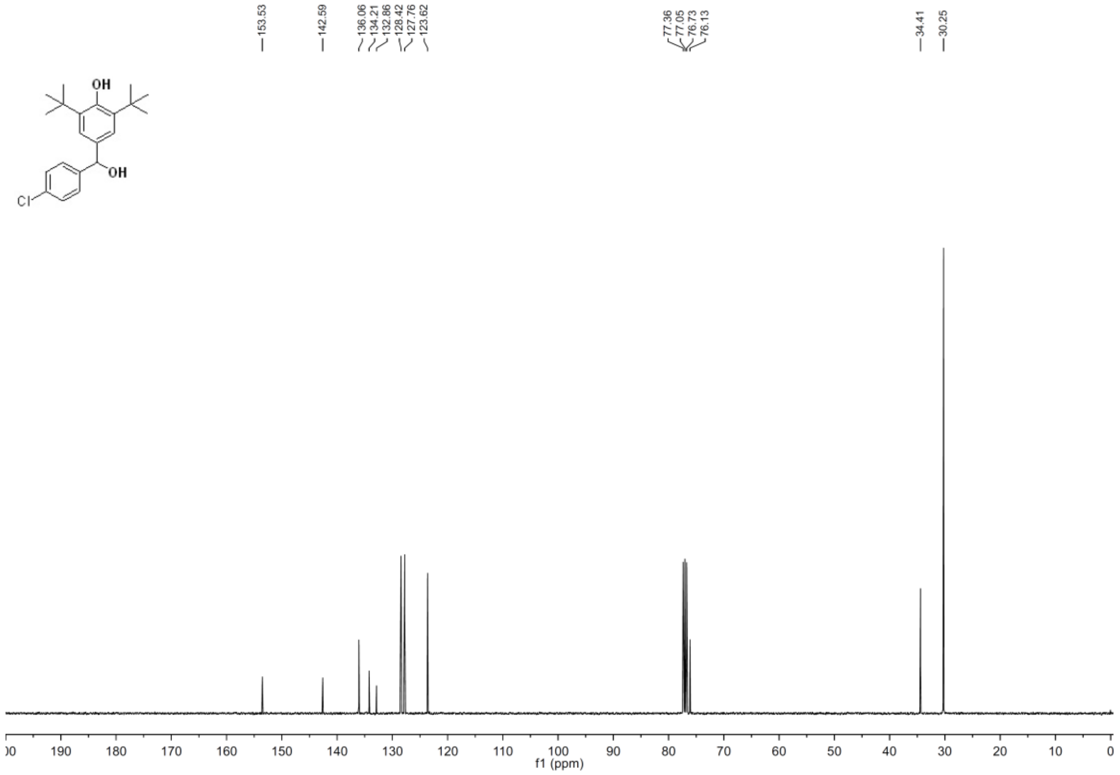

^{13}C NMR (100 MHz, $CDCl_3$) of compound **3r**

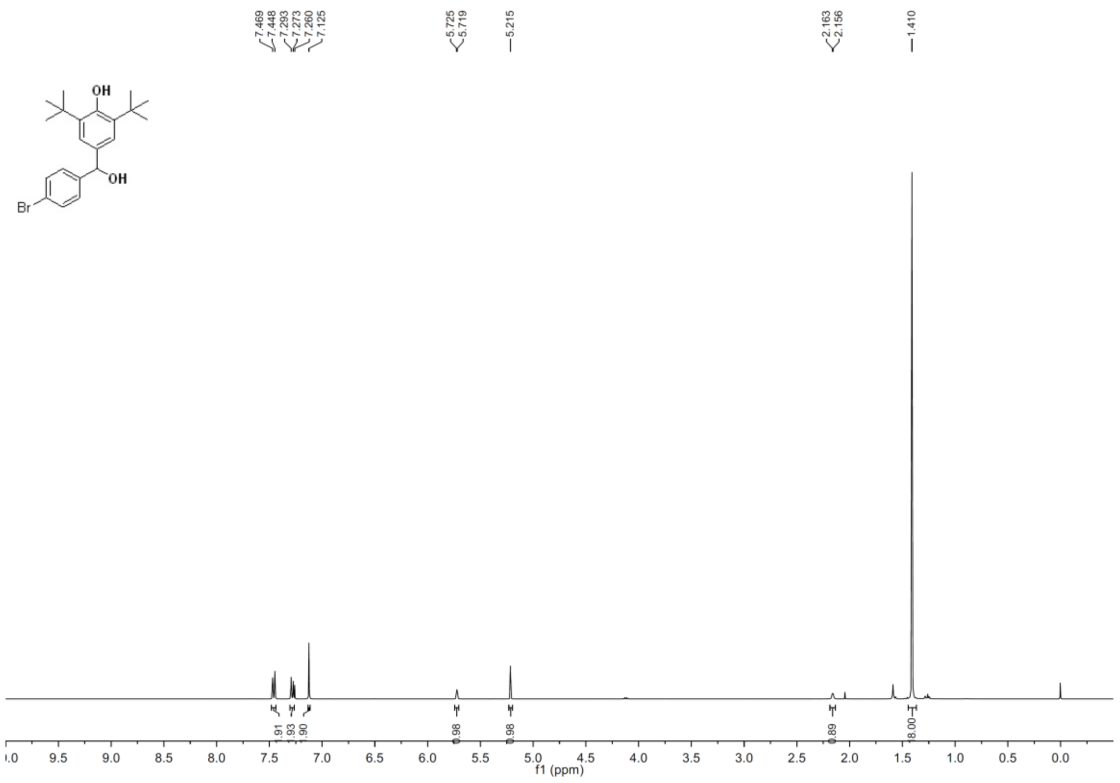

^{1}H NMR (400 MHz, $CDCl_3$) of compound **3s**

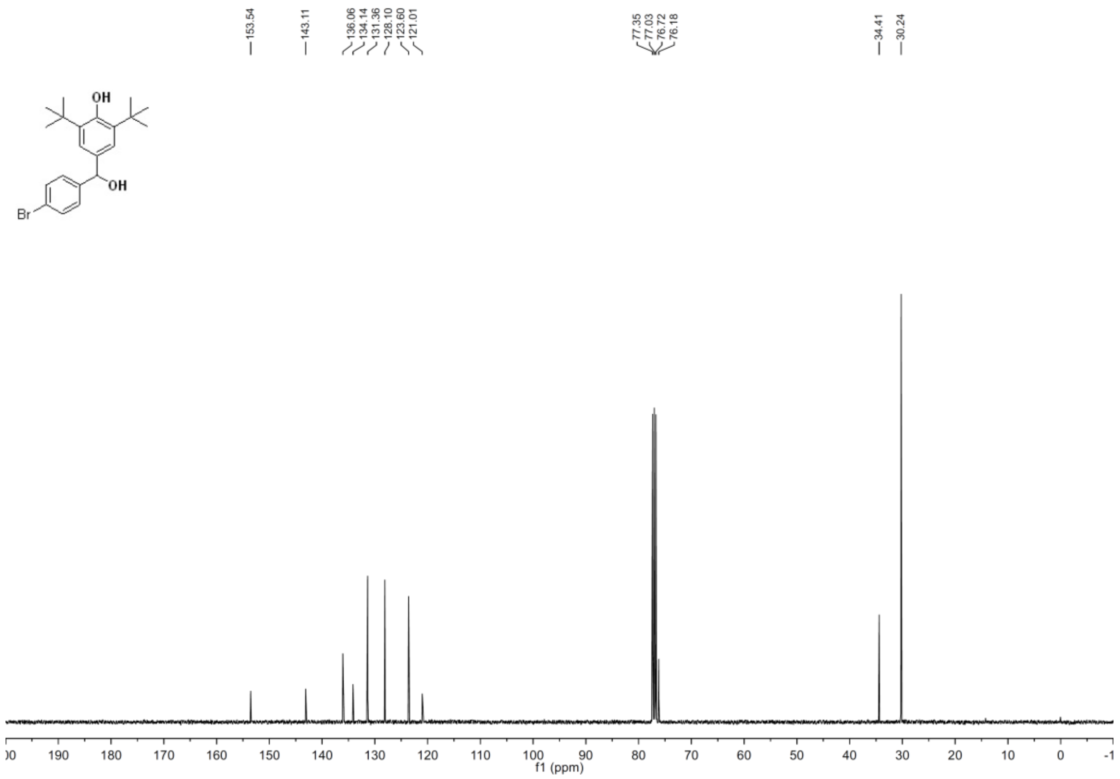

^{13}C NMR (100 MHz, $CDCl_3$) of compound **3s**

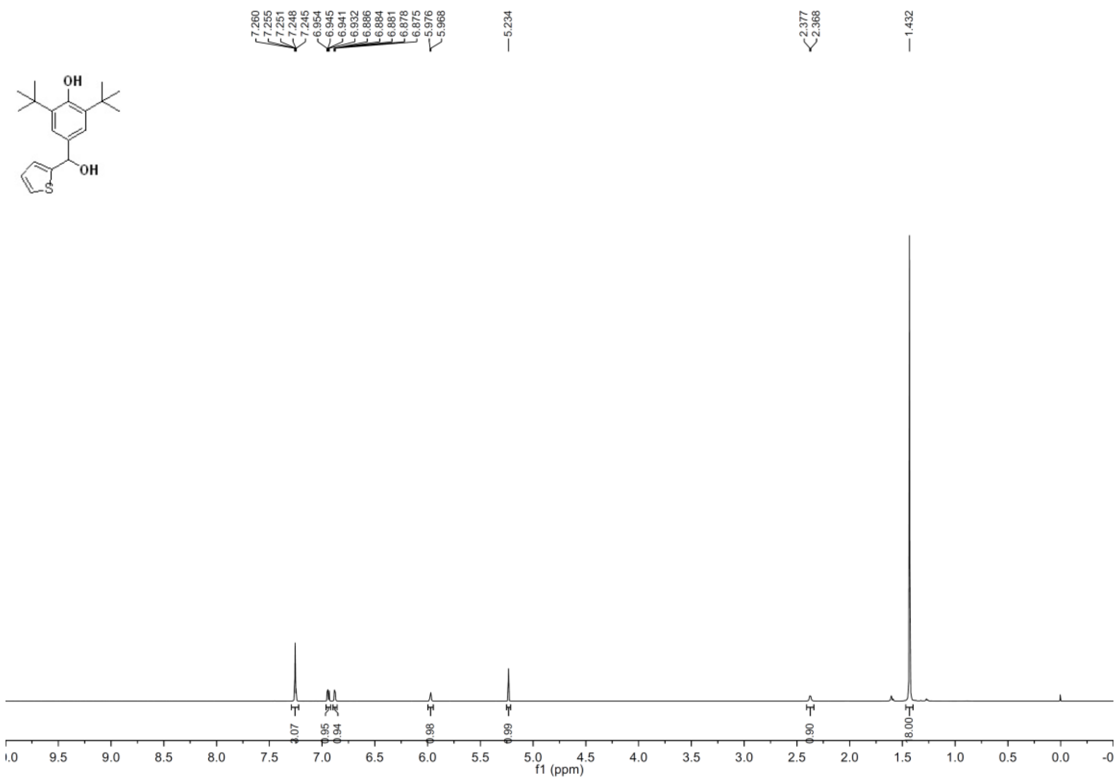

^{1}H NMR (400 MHz, $CDCl_3$) of compound **3t**

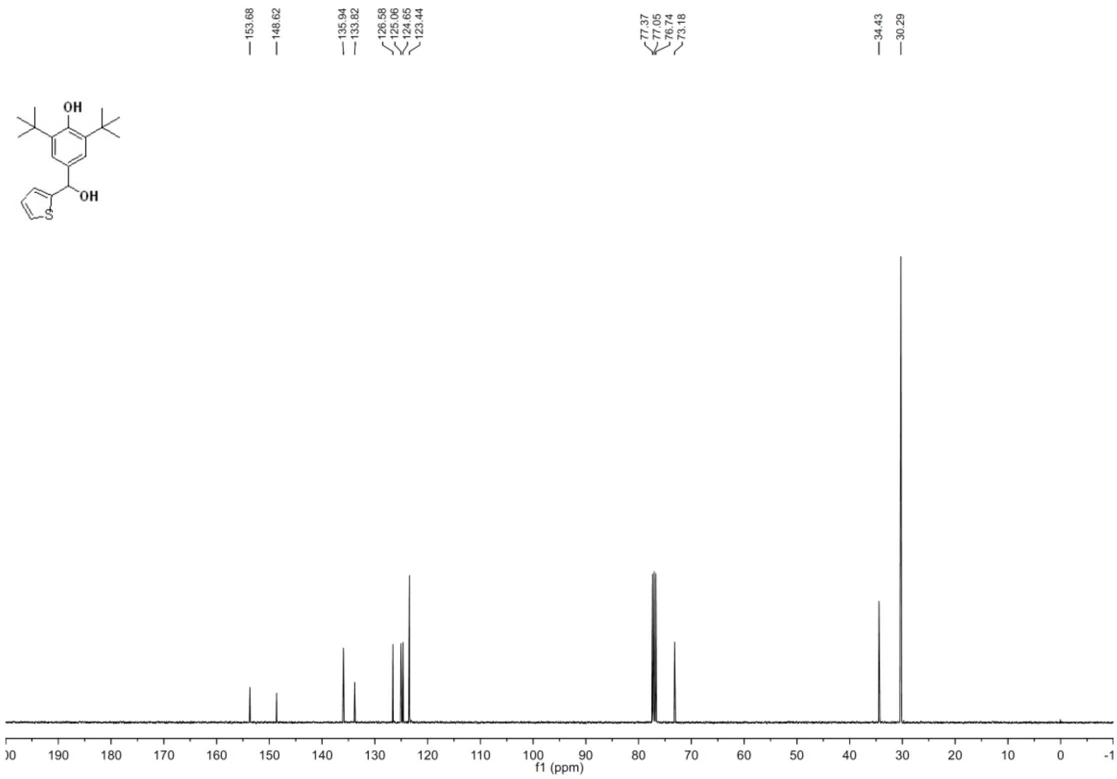

^{13}C NMR (100 MHz, $CDCl_3$) of compound **3t**
